# LncRNA ODIR1 inhibits osteogenic differentiation of hUC-MSCs through the FBXO25/H2BK120ub/H3K4me3/OSX axis

**DOI:** 10.1038/s41419-019-2148-2

**Published:** 2019-12-11

**Authors:** Shiwei He, Sheng Yang, Yanru Zhang, Xiaoling Li, Dan Gao, Yancheng Zhong, Lihua Cao, Haotian Ma, Ying Liu, Guiyuan Li, Shuping Peng, Cijun Shuai

**Affiliations:** 1 NHC Key Laboratory of Carcinogenesis, Hunan Provincial Tumor Hospital, Central South University, Changsha, 410013 China; 2 The Key Laboratory of Carcinogenesis and Cancer Invasion of the Chinese Ministry of Education, Cancer Research Institute and School of Basic Medical Sciences, Central South University, Changsha, 410078 China; 3 Hunan Key Laboratory of Non-resolving Inflammation and Cancer, Disease Genome Research Center, the Third Xiangya Hospital, Central South University, Changsha, 410013 China; 4 Department of Obstetrics and Gynecology, General Hospital, Shenzhen University, Shenzhen, 518053 China; 5 Jiangxi University of Science and Technology, Ganzhou, 341000 China; 6 State Key Laboratory of High Performance Complex Manufacturing, Central South University, Changsha, 410083 China

**Keywords:** Post-translational modifications, Mesenchymal stem cells

## Abstract

Long noncoding RNAs (lncRNAs) have been demonstrated to be important regulators during the osteogenic differentiation of mesenchymal stem cells (MSCs). We analyzed the lncRNA expression profile during osteogenic differentiation of human umbilical cord-derived mesenchymal stem cells (hUC-MSCs) and identified a significantly downregulated lncRNA RP11-527N22.2, named osteogenic differentiation inhibitory lncRNA 1, ODIR1. In hUC-MSCs, ODIR1 knockdown significantly promoted osteogenic differentiation, whereas overexpression inhibited osteogenic differentiation in vitro and in vivo. Mechanistically, ODIR1 interacts with F-box protein 25 (FBXO25) and facilitates the proteasome-dependent degradation of FBXO25 by recruiting Cullin 3 (CUL3). FBXO25 increases the mono-ubiquitination of H2BK120 (H2BK120ub) which subsequently promotes the trimethylation of H3K4 (H3K4me3). Both H2BK120ub and H3K4me3 form a loose chromatin structure, inducing the transcription of the key transcription factor osterix (OSX) and increasing the expression of the downstream osteoblast markers, osteocalcin (OCN), osteopontin (OPN), and alkaline phosphatase (ALP). In summary, ODIR1 acts as a key negative regulator during the osteogenic differentiation of hUC-MSCs through the FBXO25/H2BK120ub/H3K4me3/OSX axis, which may provide a novel understanding of lncRNAs that regulate the osteogenesis of MSCs and a potential therapeutic strategy for the regeneration of bone defects.

## Introduction

Tissue regeneration and repair through the differentiation of mesenchymal stem cells (MSCs) has been a hot topic in regenerative medicine^1–3^. Moreover, the combination of biodegradable composites with mesenchymal stem cells is expected to facilitate their use in tissue and organ repair^4–7^. MSCs have strong proliferative ability and multi-lineage differentiation, such as differentiation into fat, muscle, bone, cartilage, and other types of cells^8,9^. In particular, MSCs derived from umbilical cord (hUC-MSCs) are expected to be a new potential seed cell in tissue engineering^9,10^. The hUC-MSCs can differentiate into osteoblasts and may serve as a potential cell source for bone tissue engineering^11,12^. After hUC-MSCs incubated with osteogenic differentiation medium, the expression levels of the crucial differentiation transcription factors runt-related transcription factor-2 (RUNX2), osterix (OSX) and osteocalcin (OCN) were increased^13^.

Long non-coding RNAs (lncRNAs) are a class of RNA molecules longer than 200 nucleotides that unable to encode proteins. In recent years, increasing numbers of studies have shown that lncRNAs are involved in the osteogenesis of MSCs by regulating osteogenic transcription factors, such as RUNX2, DLX5, and OSX^14^. LncRNA ANCER inhibited the osteogenic differentiation of periodontal ligament stem cells (hPLSCs) via blocking the canonical WNT signaling pathway^15^, while lncRNA KCNQ1OT1 promoted osteogenesis through activating WNT signaling pathway^16^. LncRNA MEG3 promoted the osteogenic differentiation of MSCs by mediating BMP4 transcription activation^17^, and inhibited osteogenesis of BMSCs by suppressing miR-133a-3p^18^. LncRNA PRNCR1 increased the expression of CXCR4 through inhibiting miR-211-5p, then inhibited osteogenic differentiation and resulted in osteolysis after hip replacement^19^. Interestingly, during the human adipose-derived stem cells (hADSCs) osteogenic differentiation process, lncRNA MIR31HG promoted phosphorylation of IκBα via directly binding to IκBα and NF-κB, while the nuclear translocation of NF-κB combined to MIR31HG promoter and prompted its expression^20^. However, the roles and regulatory mechanisms of lncRNAs during hUC-MSCs osteogenic differentiation require further exploration.

In this study, we conducted a high-throughput Agilent Human lncRNA Microarray during hUC-MSCs differentiation. The lncRNA RP11-527N22.2 was significantly decreased during osteogenic differentiation of hUC-MSCs and was named osteogenic differentiation inhibitory regulator 1 (ODIR1). The biological functions and mechanism of ODIR1 in hUC-MSCs has not been reported previously. Therefore, our study demonstrates a novel ODIR1/FBXO25/OSX regulatory network regulating the osteogenic differentiation of hUC-MSCs, which may provide a potential strategy to induce osteogenic differentiation for bone regeneration.

## Results

### ODIR1 is downregulated during hUC-MSCs osteogenic differentiation

The hUC-MSCs cells were derived from human umbilical cord and its differentiation potential has been researched previously^11,12^. QC1205 is a hUC-MSCs cell line and is used in this study. The specific surface markers of the QC1205 cell line were characterized by flow cytometry, including CD44-FITC (99.7%), CD73-PE (99.8%), CD90-PerCP (99.7%), CD29-PE (99.9%), and CD34-APC (0.102%) (Supplemental Fig. 1). The expression of OSX, RUNX2 and alkaline phosphatase (ALP) were used to explore the optimum DXM concentration in osteogenic differentiation medium (OM) for hUC-MSCs, and the data showed that the optimum concentration of DXM was 100 nM (Supplemental Fig. 2A, B).

**Fig. 1:**
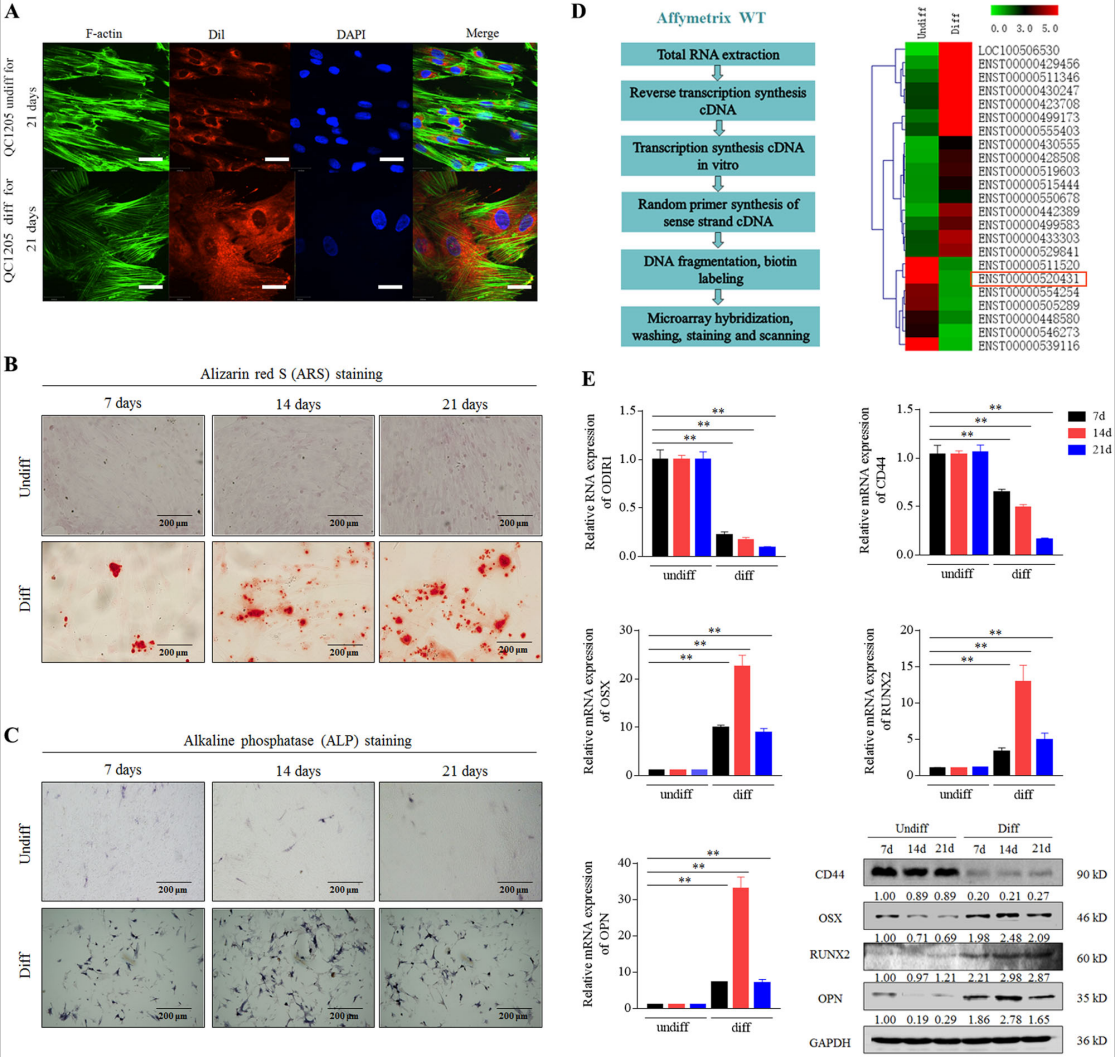
Identification of regulatory lncRNAs during osteogenic differentiation of hUC-MSCs. **a** The hUC-MSCs were cultured in normal growth medium (Undiff) or osteogenic differentiation medium (Diff) for 21 days, and analyzed by fluorescence laser confocal for cytoskeleton (F-actin), cell membrane (Dil) and nucleus (DAPI). Scale bar, 20 μm. **b**–**c** The diff and undiff hUC-MSCs cells were analyzed by Alizarin red S (ARS) and alkaline phosphatase (ALP) staining. Scale bar, 200 μm. **d** An operating flow diagram for lncRNA microarray and a heatmap for differentially expressed lncRNAs of osteogenic differentiated hUC-MSCs compared to undifferentiated hUC-MSCs. ENST00000520431 (ODIR1) was significantly decreased after osteogenic differentiation. **e** The hUC-MSCs were incubated for 7, 14, and 21 days, respectively, then the RNA levels of ODIR1, CD44, OSX, RUNX2, and OPN were analyzed by RT-qPCR, and the proteins levels of CD44, OSX, RUNX2, OPN were analyzed by western blots. GAPDH was used as internal controls.

To verify their osteogenic differentiation ability, QC1205 cells were incubated with proliferation medium (PM) or osteogenic medium (OM) for 7, 14, or 21 days, respectively. After 21 days, the morphology and cytoskeleton (F-actin staining) of the differentiation group were significantly altered, and the cell size and the nucleus became larger than those in control group (Fig. 1a,). The calcium-rich deposits and ALP activity was enhanced in 7, 14, and 21 days after osteogenic differentiation (Fig. 1b, c). Calcium nodules were analyzed by Scanning Electron Microscopy and Energy Dispersive Spectroscopy, and data suggested that calcium (Ca) and phosphorus (P) elements were increased after 28 days of osteogenic differentiation (Supplemental Fig. 3). These data confirmed that hUC-MSCs shown the potency to differentiate into osteoblasts.

To identify specific lncRNAs during osteogenic differentiation, we conducted a lncRNA profile using Agilent Human lncRNA Microarray (OE Biotech Corporation, Shanghai, China) for undiff and diff hUC-MSCs (Fig. 1d left side). The heatmap and table showed that 23 lncRNAs were significantly differentially expressed upon osteogenic differentiation (Fig. 1d and Table S1). We chose a significantly downregulated lncRNA that has not been reported previously, RP11-527N22.2, and named it lncRNA ODIR1. The RNA expression of ODIR1 was reduced in differentiated hUC-MSCs at distinct times (7, 14, and 21 days) (Fig. 1e). Moreover, the expression of the stem cells marker CD44 was significantly decreased, while the critical osteogenic transcription factors RUNX2 and OSX, and the osteogenic marker osteopontin (OPN) were obviously increased (Fig. 1e). These results demonstrated that lncRNA ODIR1 may be related to the osteogenic differentiation of MSCs.

Bioinformatic analysis indicated that ODIR1 was located on chromosome 8 and contains two exons (Supplemental Fig. 4A), with a total full length of 578 bp. ODIR1 is mainly located at the nucleus of hUC-MSCs as demonstrated by nucleoplasm separation and FISH assays (Supplemental Fig. 4B, C). In addition, the potential coding ability of ODIR1 sequences was predicted by the Coding Potential Calculator (CPC) program and the coding potential score of ODIR1 cDNA was −1.15585, which indicated that it did not have protein coding ability (Supplemental Fig. 4D). In addition, we cloned and inserted full-length ODIR1 cDNA with a HA added to its 3’ terminus (HA-ODIR1), and transfected 293 cells, with HA-GAPDH as a positive control. RT-qPCR analysis revealed that both the HA-ODIR1 and HA-GAPDH vector were expressed at high RNA levels in 293 cells, while no protein band was detected in the HA-ODIR1 group by western blotting (Supplemental Fig. 4E). Collectively, ODIR1 mainly located on nucleus and was confirmed as a non-coding RNA.

### ODIR1 inhibits the osteogenic differentiation of hUC-MSCs in vitro and in vivo

**Fig. 2:**
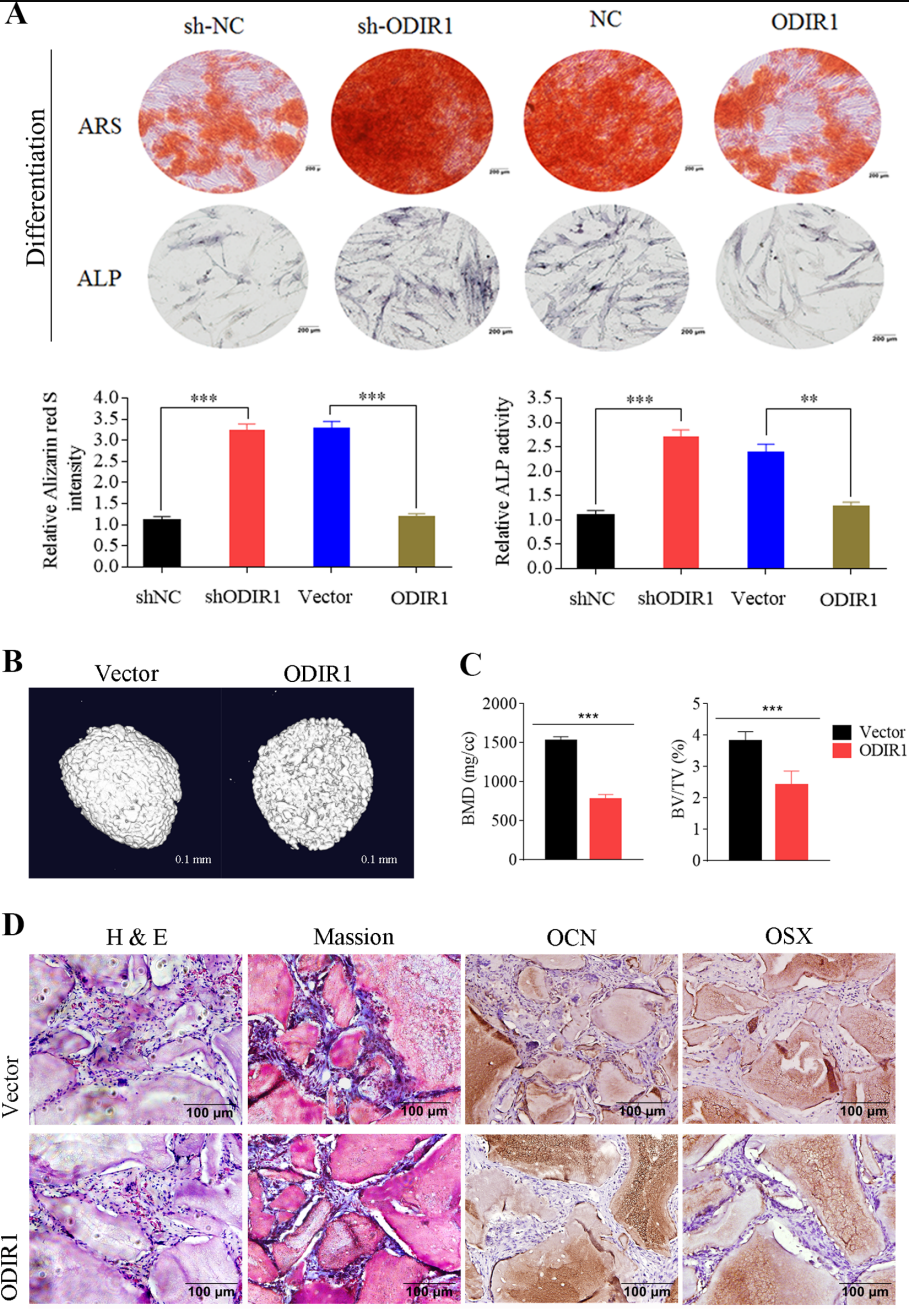
ODIR1 inhibits the osteogenic differentiation of hUC-MSCs in vitro and in vivo. **a** The hUC-MSCs were transfected with shRNA against ODIR1 or ODIR1 overexpression plasmid, following induced to differentiate into osteoblasts for 21 days. Differentiation was verified by ARS and ALP staining. **b** Representative images of bone formation (in Bio-Oss Collagen scaffolds) in ODIR1 and vector groups were detected by micro-CT. Scale bar, 0.1 mm. **c** The BV/TV (bone volume/ tissue volume, %) and BMD (bone mineral density, mg/cc) in ODIR1 and vector groups were analyzed by Xcapture Software. **d** Masson staining analyzes for collagen fiber. H and E staining analyzes for osteoid and IHC analyzes for OCN and OSX in bone matrix of ODIR1 and vector groups. Scale bar, 100 μm.

To investigate the role of ODIR1 in osteogenic differentiation, ODIR1 knockdown or ectopic expression cell lines were established by a lentiviral system. Then stable hUC-MSCs were incubated with OM for 21 days and stained using ARS or ALP kit. The results showed that the calcium nodules and ALP activity of the ODIR1 knockdown group were significantly increased (Fig. 2a).

To further verify the role of ODIR1 in the osteogenic differentiation of hUC-MSCs in vivo, a bone formation assay in nude mice was performed using Bio-Oss collagen scaffolds. After 8 weeks of osteogenic differentiation in vivo, all nude mice were sacrificed, and micro-CT analysis data suggested that less new bone formed in the ODIR1 group mice compared with the vector group (Fig. 2b). In addition, BV/TV (bone volume/tissue volume) and BMD (bone mineral density) in the ODIR1 group showed 30 and 50% decrease compared to that in the vector group, respectively (Fig. 2c). The Masson Trichrome staining results showed that less bone matrix (blue) was found in the ODIR1 overexpression group (Fig. 2d). H and E staining revealed that less newly formed bone and osteoid were observed in the ODIR1 group compared to vector group (Fig. 2d). IHC staining revealed that the intensity and range of OCN and OSX stained in osteoblasts of the ODIR1 group were less than those in the vector group (Fig. 2d), suggesting that ODIR1 inhibited the osteogenic differentiation of hUC-MSCs and the expression of OCN and OSX in vivo.

### ODIR1 inhibits the osteogenic differentiation of hUC-MSCs through OSX

**Fig. 3:**
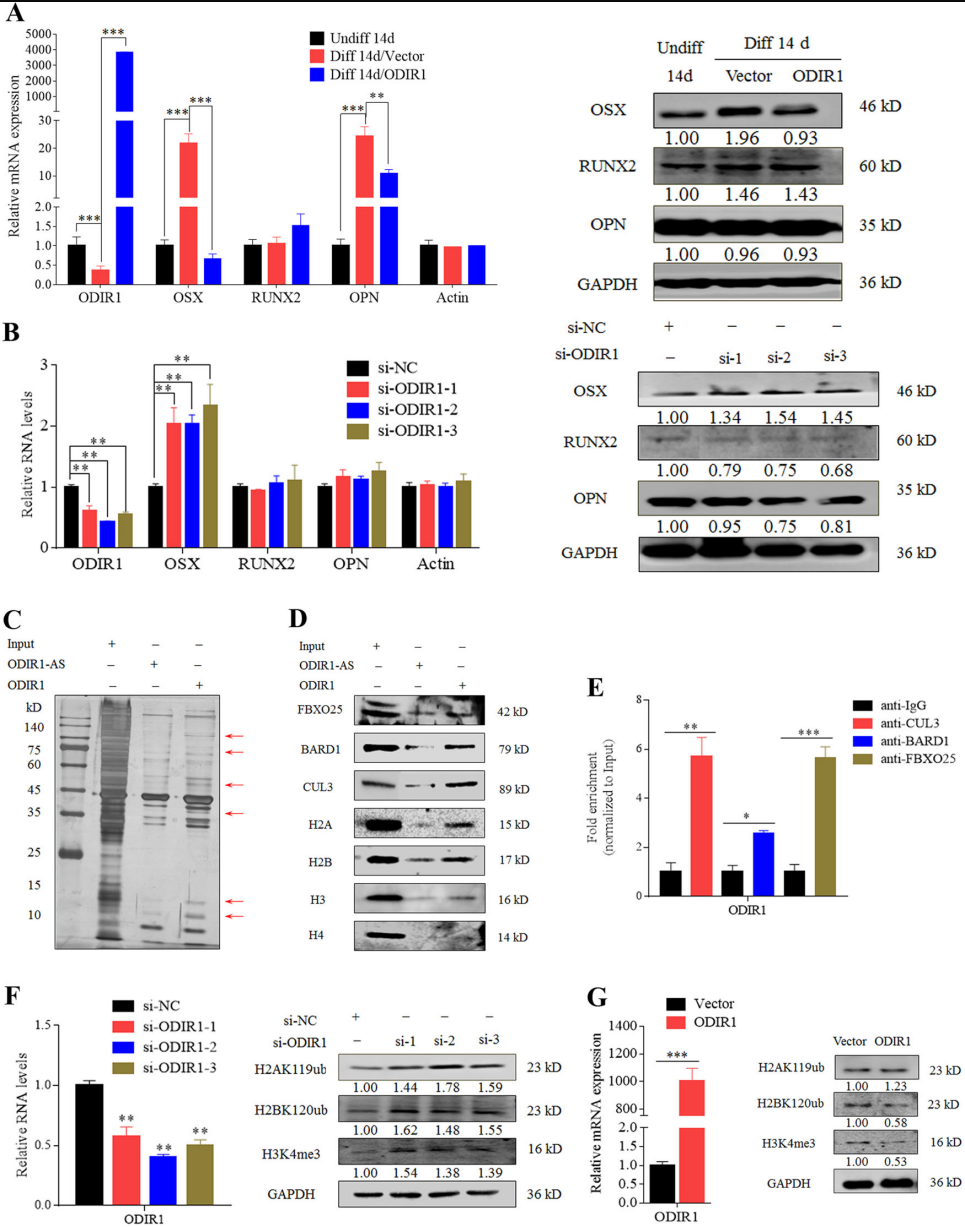
ODIR1 physically interacts with E3 ligases and histone markers. **a** The hUC-MSCs were differentiated for 14 days and transfected with vector and ODIR1 plasmid. After 24 h transfection, RT-qPCR analysis for ODIR1, OSX, RUNX2, and OPN RNA levels, western blotting analysis for OSX, RUNX2, and OPN proteins levels. **b** The hUC-MSCs were transfected with NC and ODIR1 siRNAs, RT-qPCR analysis for ODIR1, OSX, RUNX2, and OPN RNA levels, western blotting analysis for OSX, RUNX2, and OPN proteins levels. **c** Biotin-labeled ODIR1 sense and anti-sense chains were incubated with hUC-MSCs lysates, and enriched products were collected and subjected to SDS-PAGE polyacrylamide gel electrophoresis and silver staining. Differential bands were identified by LC-MS analysis. **d** ODIR1 associated with FBXO25, BARD1 and CUL3, histone proteins including H2A, H2B, H3, and H4 as shown by RNA pull-down and western blotting. **e** ODIR1 was enriched by FBXO25, BARD1, and CUL3 in hUC-MSCs lysates. Anti-IgG were used as negative control. The fold enrichment values were normalized to that of Input. **f** The hUC-MSCs were transfected with siNC and ODIR1 siRNAs, and the RNA levels of ODIR1 was measured by RT-qPCR, the proteins levels of H2AK119ub, H2BK120ub and H3K4me3 were analyzed by western blotting assay. **g** The hUC-MSCs were transfected with vector and ODIR plasmid, and the RNA levels of ODIR1, OSX, and FBXO25 was measured by RT-qPCR, the proteins levels of H2AK119ub, H2BK120ub, and H3K4me3 were analyzed by western blotting assay.

As Fig. 1e shown, the expression of the crucial osteogenic transcription factors RUNX2 and OSX were increased, while ODIR1 expression was decreased during the osteogenic differentiation of hUC-MSCs. To explore whether ODIR1 regulates the expression of OSX or RUNX2, we performed RT-qPCR and western blotting after ODIR1 ectopic expression or knockdown. After ODIR1 overexpression, OSX mRNA and protein levels were significantly reduced, while RUNX2 was not altered in the osteogenic differentiation of hUC-MSCs (Fig. 3a). Correspondingly, the OSX mRNA and protein were significantly increased in the ODIR1 knockdown group, while RUNX2 was also not altered in hUC-MSCs (Fig. 3b).

To further determine how ODIR1 functions in the osteogenic differentiation of hUC-MSCs through OSX, RNA pulled down assay and mass spectrometry analyses were performed. The pull-down proteins were subjected to SDS-PAGE electrophoretic analysis, and the differential bands were analyzed by Mass Spectrum analysis (MS) (Fig. 3c). The MS results suggested that ODIR1 physically interacted with multiple proteins (Table S2). The GO functional annotation of these proteins is indicated in Table S3, and the data suggested that these proteins mainly involved in exocytosis, secretion and export from cells, single-stranded RNA and DNA binding. Epigenetic modification of histone H2A, H2B, H3, and H4 proteins is associated with gene transcription, and histone mark H2B mono-ubiquitination at lysine 120 (H2BK120ub) increased the level of H3 trimethylation at lysine 4 (H3K4me3)^21,22^. Further western blotting assay verified the ODIR1 pull-down proteins in hUC-MSCs lysates (Fig. 3d). RIP assays also confirmed the physical association between FBXO25, BARD1 or CUL3, and ODIR1 (Fig. 3e). Taken together, ODIR1 physically associates with the E3 ligases FBXO25, BARD1, CUL3 and the histone proteins H2A, H2B, and H4.

### ODIR1 inhibits OSX transcription by altering the enrichment of H2BK120ub and H3K4me3 on the OSX promoter region

**Fig. 4:**
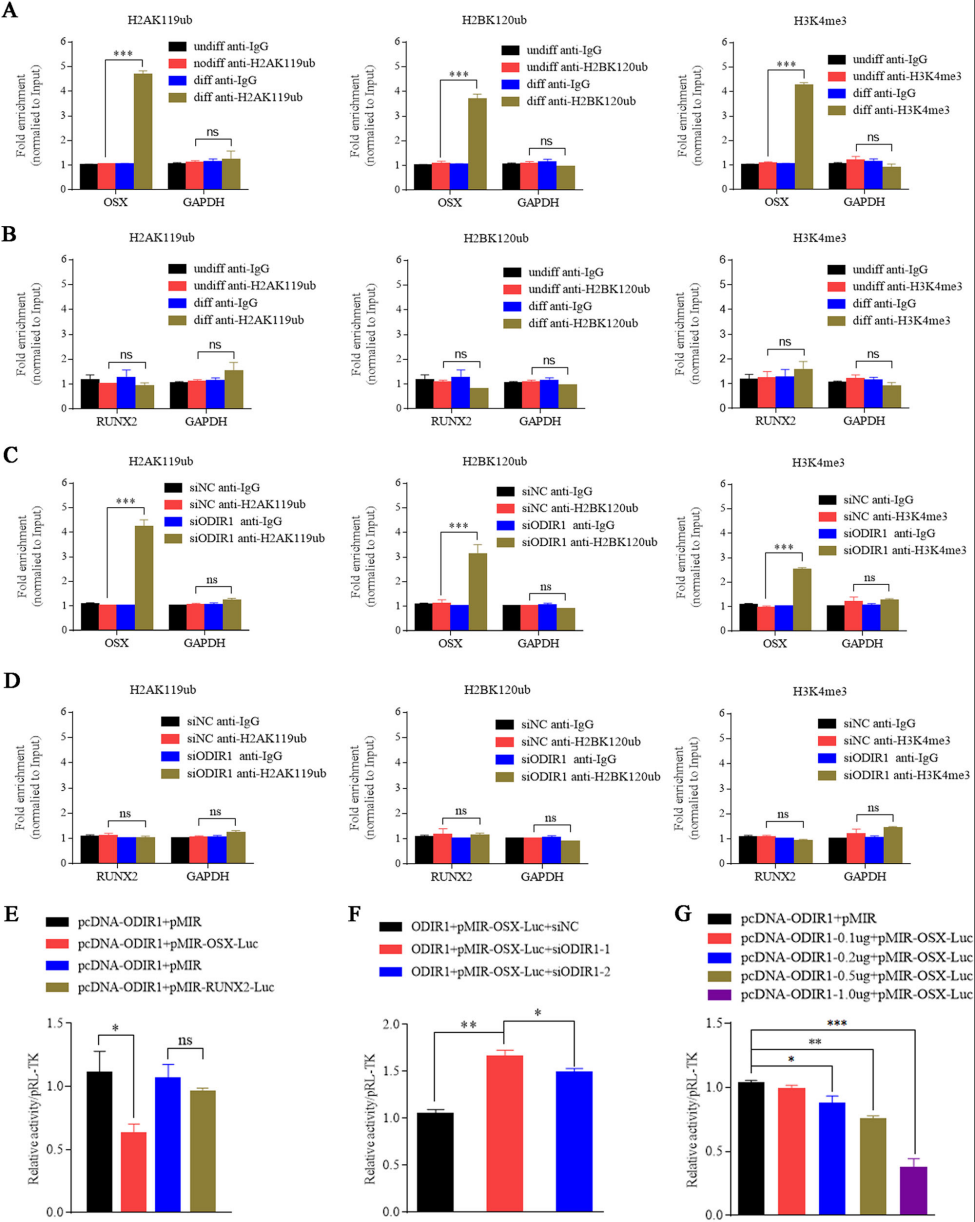
ODIR1 alters the modification of histone marks on OSX promoter. **a**, **b** The hUC-MSCs were cultured in normal growth medium (Undiff) or in osteogenic differentiation medium (Diff) for 10 days. The antibodies H2AK119ub, H2BK120ub, H3K4me3, and IgG were incubated with cell lysates, respectively, and the enrichment of those three histone markers on OSX or RUNX2 promoter was analyzed by ChIP-qPCR. **c**, **d** The hUC-MSCs were transfected with NC and ODIR1 siRNAs. The antibodies H2AK119ub, H2BK120ub, H3K4me3, and IgG were incubated with cell lysates, respectively, and the enrichment of those three histone markers on OSX or RUNX2 was analyzed by ChIP-qPCR. **e** The pMIR-OSX-luc (containing OSX promoter region) or pMIR-RUNX2-luc (containing RUNX2 promoter region) plasmids were co-transfected with pCDH-ODIR1 or pCDH plasmids into 293 cells, respectively, and the cell lysates were analyzed by luciferase assay. **f** The pMIR-OSX-luc plasmid was co-transfected with NC and ODIR1 siRNAs into 293 cells, respectively, and the cell lysates were analyzed by luciferase assay. **g** The pMIR-OSX-luc plasmids were co-transfected with different amounts (0.1, 0.2, 0.5, 1.0 μg) of pCDH-ODIR1 plasmids into 293 cells, respectively, and analyzed by luciferase assay.

ODIR1 knockdown increased the levels of H2AK119ub, H2BK120ub, and H3K4me3 (Fig. 3f), while ODIR1 overexpression decreased the expression of those proteins (Fig. 3g). It is known that lncRNAs regulate gene expression related to cellular activities through transcriptional or post transcriptional regulation^23^. H2BK120 ubiquitination can lead to transcriptional activation of target genes^24^, and it also promotes the tri-methylation of histone H3 at lysine 4 (H3K4me3). H3K4me3 modification was enriched on activated promoters and promotes the transcription of target gene^25^. Therefore, we first examined whether ODIR1 caused the fold enrichment of these histone epigenetic marks at the OSX or RUNX2 promoter region. Firstly, hUC-MSCs were incubated with OM for 7 days and the RNA levels of ODIR1 were decreased (Supplemental Fig. 5A), and the ChIP-qPCR results showed that low ODIR1 expression led to a significant increase enrichment of histone marks H2AK119ub (4.6 fold), H2BK120ub (3.7 fold), and H3K4me3 (4.3 fold) at the OSX but not at the RUNX2 promoter region (Fig. 4a, b). Moreover, knockdown of ODIR1 in hUC-MSCs (Supplemental Fig. 5B) also resulted in an increase in fold enrichment of H2AK119ub (4.2 fold), H2BK120ub (3.2 fold), and H3K4me3 (1.6 fold) at the promoter region of OSX but not RUNX2 in hUC-MSCs (Fig. 4c, d). These data showed that ODIR1 inhibited osteogenesis by regulating OSX rather than RUNX2 in transcription level.

To further confirm the role of ODIR1 in the transcription of OSX, we detected the luciferase activities of the OSX promoter region using a dual luciferase reporter assay. The luciferase activity of the OSX promoter was markedly decreased while the activity of the RUNX2 promoter was not altered in the ODIR1 overexpression group (Fig. 4e). The luciferase activity of the OSX promoter also increased in the ODIR1 knockdown group (Fig. 4f). In addition, we found the luciferase activity of OSX promoter decreased in an ODIR1 dose-dependent pattern (Fig. 4g).

### ODIR1 inhibits H2B mono-ubiquitination through FBXO25 ubiquitination

**Fig. 5:**
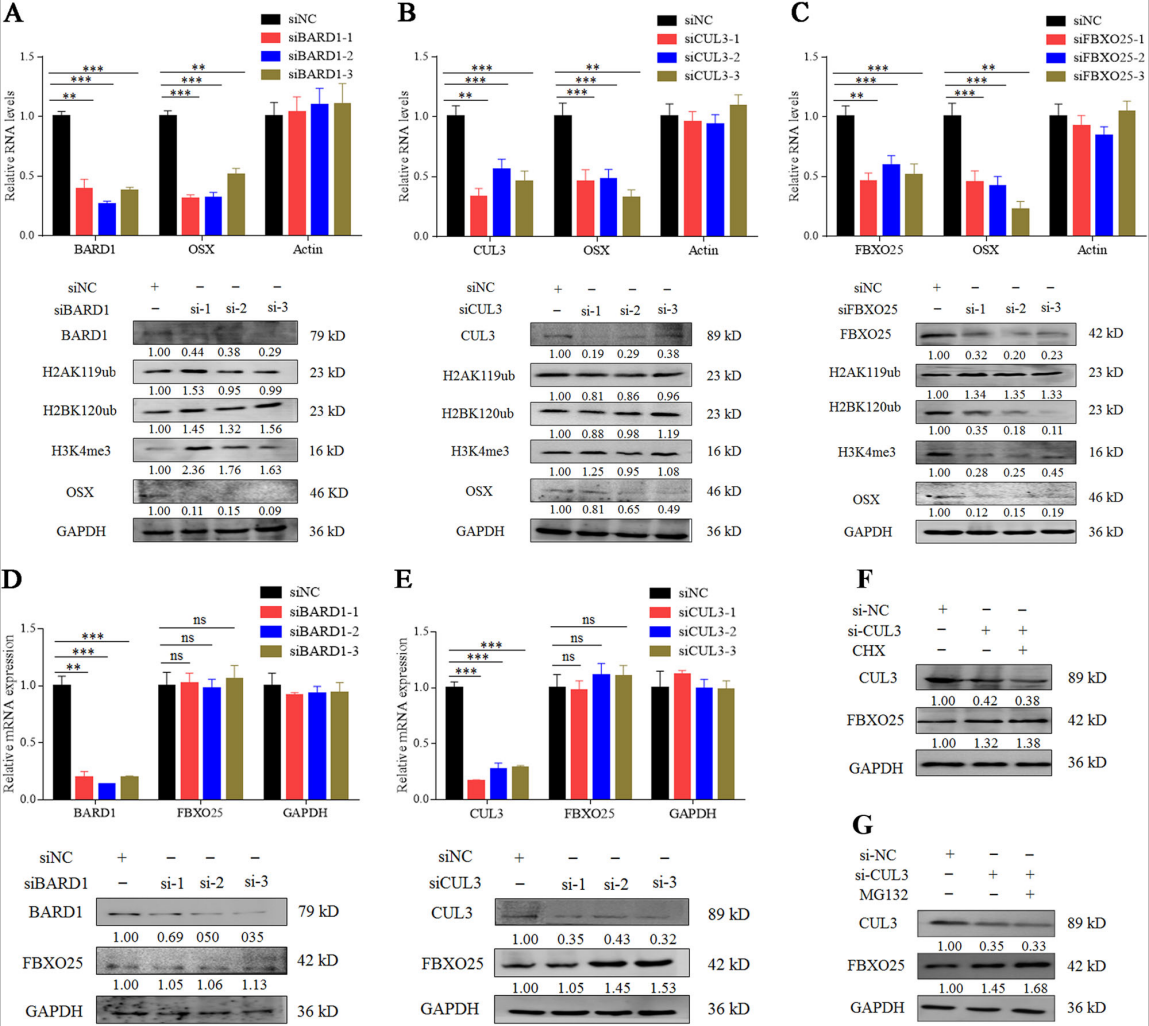
FBXO25 promotes the mono-ubiquitination of H2BK120 and tri-methylation of H3K4 in hUC-MSCs. **a** The hUC-MSCs were transfected with NC and BARD1 siRNAs, RT-qPCR was performed for detecting the RNA levels of BARD1 and OSX, western blotting was conducted for detecting the proteins levels of BARD, H2K119ub, H2BK120ub, H3K4me3 and OSX. **b** The hUC-MSCs were transfected with NC and CUL3 siRNAs, RT-qPCR was performed for detecting the RNA levels of CUL3 and OSX, western blotting was conducted for detecting the proteins levels of CUL3, H2K119ub, H2BK120ub, H3K4me3, and OSX. **c** The hUC-MSCs were transfected with NC and FBXO25 siRNAs, RT-qPCR was performed for detecting the RNA levels of FBXO25 and OSX, western blotting was conducted for detecting the proteins levels of FBXO25, H2K119ub, H2BK120ub, H3K4me3, and OSX. **d** The hUC-MSCs were transfected with NC and BARD1 siRNAs, and the proteins levels of BARD1 and FBXO25 were analyzed by western blotting assay. **e** The hUC-MSCs were transfected with NC and CUL3 siRNAs, and the proteins levels of CUL3 and FBXO25 were analyzed by western blotting assay. **f** and **g** The hUC-MSCs transfected with siNC or siCUL3 for 24 h, then treated with CHX and MG132, respectively. The protein synthesis and stability of FBXO25 affected by CUL3 were determined by western blotting.

To explore the definite mechanism of H2BK120 mono-ubiquitination, we explored the relationship among the above three E3 ligases (FBXO25, CUL3, and BARD1) and H2AK119ub, H2BK120ub, and H3K4me3. Firstly, after hUC-MSCs were cultured in OM for 7, 14, and 21 days, the natural histones H2A, H2B and H3 were not significantly altered, while the modified status H2AK119ub, H2BK120ub, and H3K4me3 were upregulated (Supplemental Fig. 6A). The proteins level of FBXO25 was gradually increased (Supplemental Fig. 6A). FISH analysis suggested that ODIR1 and FBXO25 mainly co-located at cell nucleus (Supplemental Fig. 6B). Moreover, ODIR1 decreased the protein levels of FBXO25 and CUL3 but did not affect BARD1 protein levels (Supplemental Fig. 6C, D). After transfection with specific siRNAs against BARD1, CUL3 and FBXO25, the expression of OSX was decreased while H2AK119ub was not changed; only knockdown of FBXO25 decreased the levels of H2BK120ub and H3K4me3 (Fig. 5a-c). FBXO25 (F-box protein 25), a member of the F-box protein family, is one of the four subunits of the SKP1-cullin-F-box (SCF) ubiquitin ligase complex^26^. CUL3 (Cullin 3), an E3 ubiquitin ligase, mediates the ubiquitination of target proteins such as H2A, through the Cullin-RING-based BCR complex^27^. Another E3 ligase of the Cullin family, Cul4, which forms a Clr4-Rik1-Cul4 complex and recruits H2B and H4 to form RNAi-mediated heterochromatin^28^. BARD1 (BRCA1 associated RING domain 1) and BRCA1 forms an E3 ubiquitin-protein ligase heterodimer that mediated H2A/H2B ubiquitination and regulated the differentiation of mesenchymal stem cells^29^. In conclusion, CUL3, BARD1, and FBXO25 are associated with the ubiquitination of histone proteins. In addition, we found that knockdown of BARD1 and CUL3 did not affect the RNA levels of FBXO25 while knockdown of CUL3 increased the protein level of FBXO25 (Fig. 5d, e). Given that CUL3 is an E3 ligase, we next determined whether CUL3 could reduce FBXO25 protein levels by promoting its ubiquitination and degradation. As expected, CUL3 promoted FBXO25 degradation through the proteasome pathway (Fig. 5f, g).

**Fig. 6:**
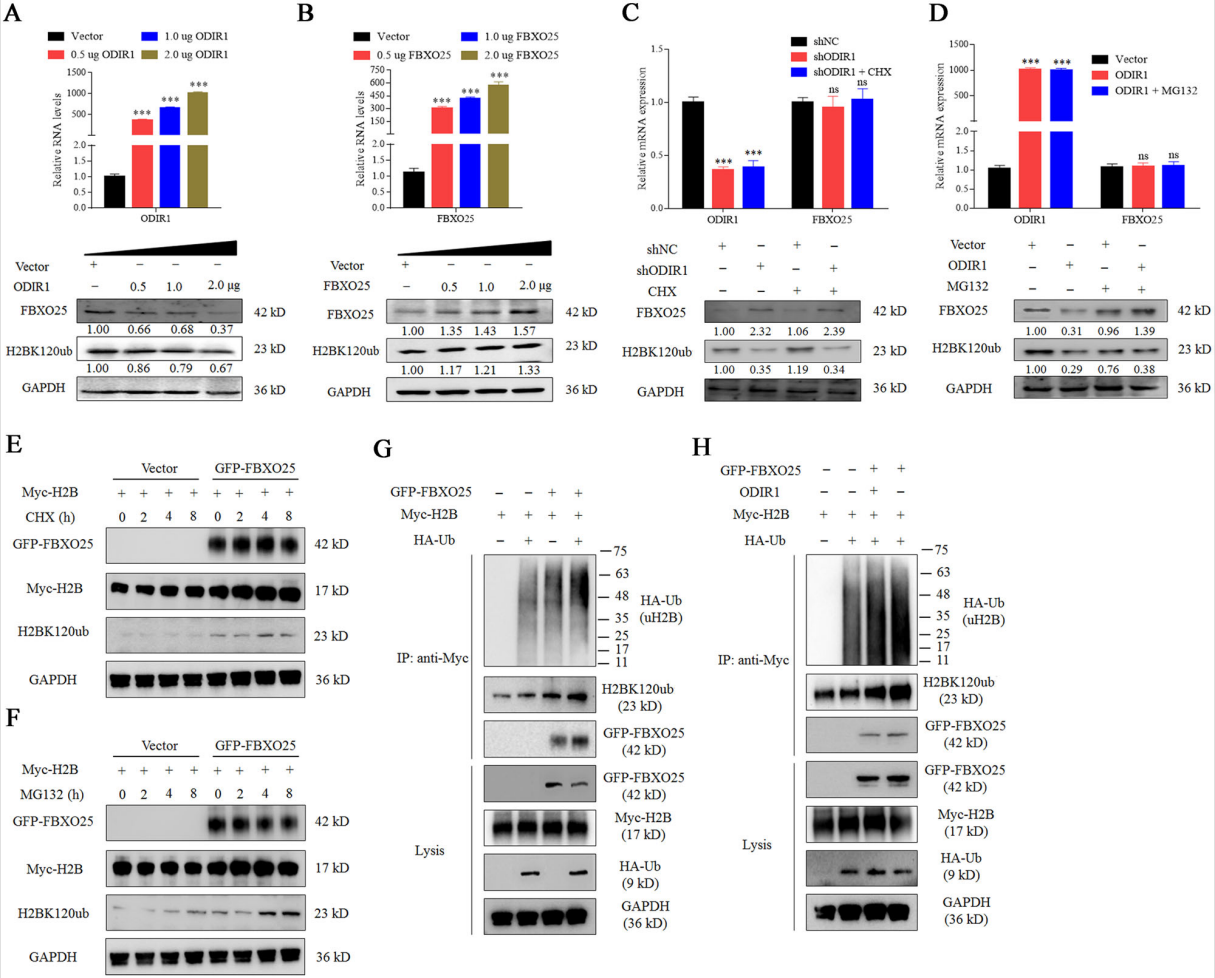
ODIR1 restrains the levels of H2BK120ub protein through recruiting FBXO25. **a** The hUC-MSCs were transfected with vector and different amounts (0, 0.5, 1.0, and 2.0 μg) of ODIR1 plasmid, the RNA levels of ODIR1 was measured by RT-qPCR and the proteins levels of FBXO25 and H2BK120ub were detected by western blotting. **b** The hUC-MSCs were transfected with vector and different amounts (0, 0.5, 1.0, and 2.0 μg) of FBXO25 plasmid, the RNA levels of FBXO25 was measured by RT-qPCR and the proteins levels of FBXO25 and H2BK120ub were detected by western blotting in cell lysates. **c** The hUC-MSCs were transfected with shCtrl or shODIR1 plasmid and treated with CHX, and the proteins levels of FBXO25 and H2BK120ub were analyzed by western blotting assay. **d** The hUC-MSCs were transfected with vector or ODIR1 plasmid and treated with MG132, and the proteins levels of FBXO25 and H2BK120ub were analyzed by western blotting assay. **e** The HEK293T cells were transfected with FBXO25 overexpression vector, followed by CHX treatment for indicated periods of time and then H2BK120ub were detected by western blot. **f** The HEK293T cells were transfected with FBXO25 overexpression vector, followed by MG132 treatment for indicated periods of time and then H2BK120ub were detected by western blot. **g** HEK293T cells were transfected with GFP-FBXO25, Myc-H2B and HA-Ub as indicated. After 48 h culture, the levels of ubiquitinated H2B were monitored by IP analysis, followed by western blot with indicated antibodies. **h** HEK293T cells were transfected with ODIR1, GFP-FBXO25, Myc-H2B, and HA-Ub as indicated. After 48 h incubation, the levels of ubiquitinated H2B were monitored by IP analysis, followed by western blot with indicated antibodies.

In addition, as the amount of ODIR1 transfection increased, the inhibition of FBXO25 and H2BK20ubi increased gradually; as the amount of transfection of FBXO25 increases, the protein levels of FBXO25 and H2BK20ubi also increase gradually. Moreover, ectopic expression of ODIR1 reduced the ubiquitination of H2BK120 protein and shortened the H2BK120ub protein half-life (Fig. 6c, d). Ectopic expression of FBXO25 did not affect the expression or half-life of exogenous H2B protein but increased the protein levels of H2BK20ubi (Fig. 6e, f). We then performed a denaturing immunoprecipitation assay to investigate whether FBXO25 affects the ubiquitination levels of H2BK120ub. The data revealed that FBXO25 increased the levels of H2BK120ub (Fig. 6g), however, the increase of H2BK120ub was obviously reversed by ODIR1 overexpression (Fig. 6h). These results indicate that ODIR1 inhibits FBXO25-mediated H2B ubiquitination.

## Discussion

MSCs differentiation into osteoblasts is a multiple-step and extremely complicated process, and the BMP-Smad signaling pathway is one of the regulatory axis in this process^30^. When bone morphogenetic protein (BMPs) is activated and targets Smad family members, SMADs are phosphorylated and sequentially facilitated the transcription of the osteogenesis factors RUNX2 and OSX and then activated the expression of downstream osteoblast markers, such as OCN, OPN, and Col1al^31,32^. In this process, RUNX2 mediated MSCs differentiation into osteochondro-progenitors and simultaneously activated OSX and ATF4 during the osteochondro-progenitor differentiation into immature osteoblasts, mature osteoblasts and finally matured into osteocytes^31–33^. RUNX2 (CBFA; core-binding factor-alpha) is a member of the runt-homology domain protein family^34^. OSX is an essential transcription factor contains a zinc-finger domain^32^. Phosphorylation of RUNX2 activated the transcription of OSX during osteogenesis of stem cells^35^. In this study, we found that lncRNA ODIR1 downregulated while both RUNX2 and OSX upregulated during the osteogenic differentiation of hUC-MSCs (Fig. 1e).

As shown, ODIR1 could recruit FBXO25, BARD1, and CUL3, while only FBXO25 and CUL3 could regulate the levels of H2BK120ub and H3K4me3, which suggested that FBXO25 and CUL3 might be involved in histone modification (Fig. 3c, d). FBXO25 is a member of the F-boxing protein (FBP) family contains an ubiquitination target binding domain, and is one subunit of SCFs (Skp1-Cul1-FBP) complex^26^. FBP is responsible for recruiting substrates to SCF complex, and then binding to the E3 ligase members SCF, Skp1, and Cul1; finally, ubiquitinating specific substrates under physiological or pathological process^36^. It has been found that FBXO25 acts as a novel E3 ligase to ubiquitinate the cardiac transcription factors Isl1, Nkx2-5, and Hand1, and is involved in hypertrophic cardiomyocyte growth and cardiogenesis^37^. In addition, FBXO25 decreased the active levels of phosphorylated ERK1/2 through its E3 ligase SCF1 activity^38^. As an E3 ubiquitin ligase, FBXO25 promotes proteins degradation mainly through the ubiquitination pathway, while the mechanism by which FBXO25 regulates histone marks through epigenetic modification was elusive. In this study, we found that FBXO25 increased the mono-ubiquitination of H2BK120 and subsequently the tri-methylation of H3K4 during osteogenesis (Fig. 5c), which indicated that FBXO25 regulates the histone modification during hUC-MSCs differentiation.

During the osteogenic differentiation of WJ-MSCs, the transcription of RUNX2 is strongly activated, and that of OSX is inactivated. The mechanism suggested that high activity of the activating histone marks H3K27Ac, H3Ac, H3K4me1 and H3K4me3 occupancies on RUNX2 promoter region, while the high activity of inhibitory histone marks H3K27me3 and H3K9me3 was enriched at the promoter region of OSX^39^. In our study, we found that the activities of H2AK119ub (histone H2A mono-ubiquitinated at lysine 119), H2BK120ub (histone H2A monoubiquitinated at lysine 120) and H3K4me3 (histone H3 trimethylated at lysine 4) were significantly increased during the osteogenic differentiation of hUC-MSCs (Fig. S6A). In mammalian cells, H2A mono-ubiquitination is mediated by polycomb repressive complex 2 (PRC2) and is related to transcriptional repression^40,41^. CRL4B (Cullin4B-Ring E3 ligase complex) physically interacts with PRC2 and then attenuates the activity of H2AK119ub and H3K27me3 (transcriptional inactivated marks)^42^. Contrary to H2AK119, H2BK120ub is an activating histone mark, and ubiquitination is mediated by the ubiquitin-conjugating enzyme RAD6^40,43^. H2BK120ub attenuates MEIS1-mediated apoptosis^44^ or maintains the pluripotency of stem cells by promoting the expression of pluripotential genes^45^. During mouse embryonic stem cells (ESCs) differentiation, H2BK120ub was preferentially abounded on the coding region of differentiation-related transcription factors OCT4, Sox17, Nanog, etc, and the decrease in H2BK120ub affects their transcription, triggering further transforms in ESC differentiation^46^. High expression of USP44 maintains the low levels of H2Bubi in undifferentiated mouse ESCs; when cells in an efficient differentiated state, USP44 levels decreased while H2BK120ub increased. Mechanistically, depletion of USP44 prior to the differentiation signal promotes H2Bubi expression; however, impairing the dynamic turnover of H2BK120ubinterferes with proper execution of the differentiation process^47^. In eukaryotes, H3K4me3 is an activating histone mark that is associated with gene expression and active chromatin, and can be methylated (mono-methylated, di-methylated, or tri-methylated) by SET7 or demethylated by JARID1A^48,49^. Several studies have reported that H2B mono-ubiquitination can facilitate H3K4 trimethylation on chromatin. H2BK120ub mediated by Set1-COMPASS can promote the activities of H3K4me3^50^. hRAD6ubiquitylates H2BK120 on chromatinized via direct interaction with hPAF-bound hBRE1and the latter directly stimulated H3K4 di-methylation or tri-methylation^21^. During myogenic differentiation, the RNF20 mediated histone talk between H2BK120ub and H3K4me3 is decreased, which demonstrated that Set1-dependent H3K4me3 was inhibited by the decreased interaction with H2BK120ub^22^. In this study, we found that ODIR1 reduced the occupancies of H2BK120ub and H3K4me3 on the OSX promoter (Fig. 4). In ODIR1 knockdown hUC-MSCs, we found increased occupancy of inhibitory mark H2AK119ub at the OSX promoter, while the transcription of OSX was increased instead of inhibited, which suggested that the activation effect of H2BK120ub coordinate with H3K4me3 on the OSX promoter region may be slightly stronger than the inhibition effect of H2AK119ub. According to the experimental results, during osteogenic differentiation, OSX is the early osteogenic transcription factor during osteochondro progenitor differentiation into immature osteoblasts and the subsequent osteoblasts maturation process. In summary, the dynamic changes in OSX level might contribute to the multi-step osteogenic differentiation process of MSCs.

**Fig. 7:**
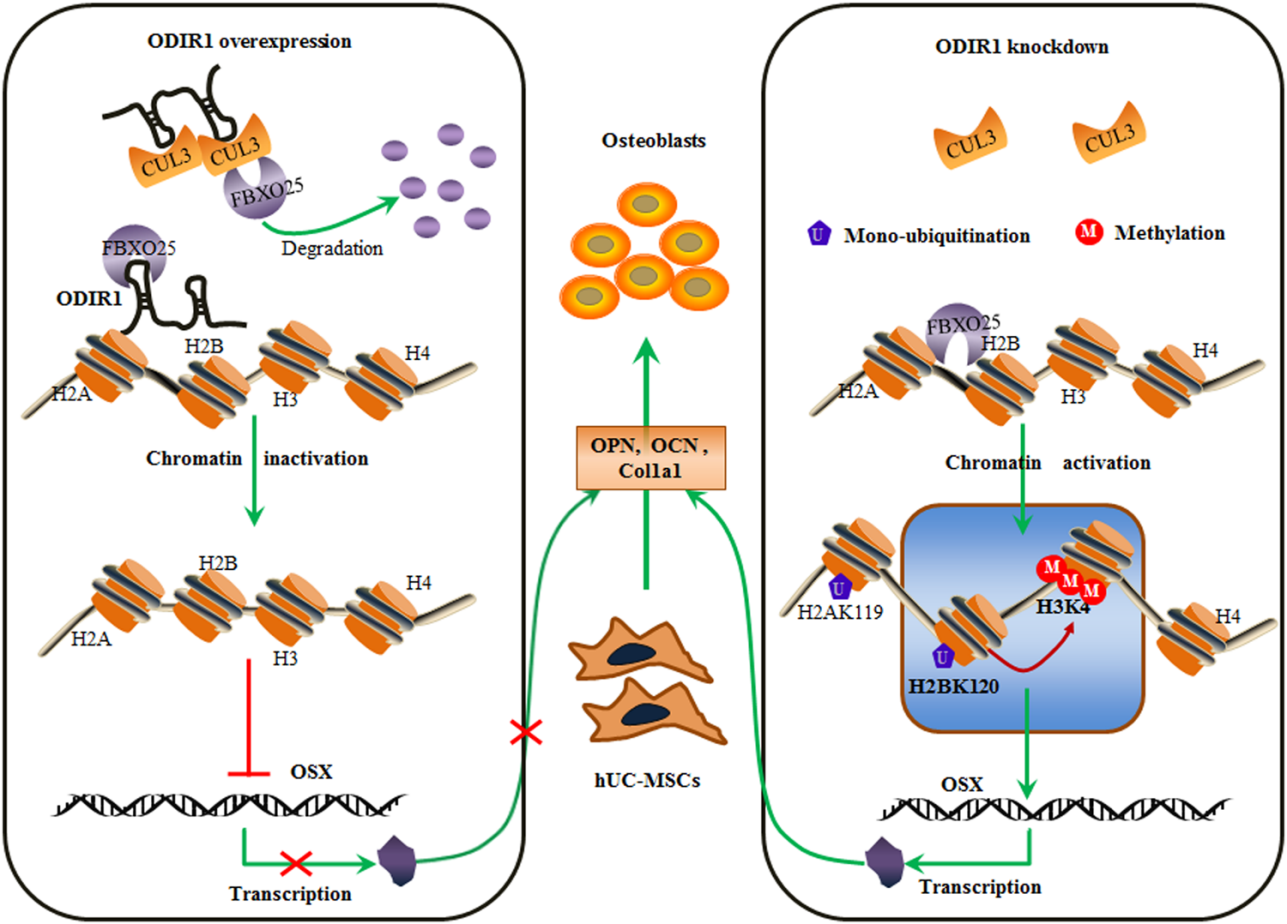
A work model of ODIR1 during hUC-MSCs osteogenic differentiation. In this model, in the UC-MSCs with no induction, ODIR1 RNA is high and recruits E3 ubiquitin ligase CUL3,BARD1 and FBXO25, FBXO25 was degraded by CUL3 complex, which lead to low levels of H2BK120 mono-ubiquitination (H2BK120ub) and H3K4 trimethylation (H3K4me3), and the transcription levels of OSX would be repressed; In the UC-MSCs with osteogenic induction or those with ODIR1 knockdown, high level of FBXO25 promotes H2BK120 mono-ubiquitylating (H2BK120ub) which following stimulates H3K4 trimethylation (H3K4me3), and the transcription levels of OSX would be activated, the increased proteins expression of OSX promotes hUCMSCs differentiating into osteoblasts.

LncRNAs are involved in epigenetic regulation by recruiting related essential molecules to the methylated or ubiquitinated protein, and then exerts their biological functions via transcriptional activation or repression^51^. Overall, we propose a tentative work model of ODIR1 during osteogenic differentiation of hUC-MSCs (Fig. 7). When ODIR1 expression is high level in hUC-MSCs, it recruits the E3 ubiquitin ligase CUL3, which induces the degradation of FBXO25 and then suppresses the levels of H2BK120ub and subsequently H3K4me3 occupying on the OSX promoter region. After ODIR1 knockdown in hUC-MSCs, the decoy and inhibitory effects of ODIR1 on FBXO25 and H2BK120 are released, thus inducing H2BK120 mono-ubiquitination and subsequent H3K4 tri-methylation enrichment on the OSX promoter region The transcription of OSX increases and promotes the transcription of downstream osteogenic genes, such as OPN, OCN, and Col1a1, ultimately promoting hUC-MSCs differentiation into osteoblasts.

## Materials and methods

### Cell culture and osteogenic induction

The human umbilical cord-derived mesenchymal stem cell (hUC-MSCs) line QC1205 was obtained from the National Engineering Research Center of Human Stem Cells (Changsha, China) and maintained at 37 °C in DME/F12 (1:1) medium with 10% FBS (Gibco, Grand Island, NT, USA). The QC1205 cell line was characterized using flow cytometry (BD, New Jersey, USA) for MSCs specific surface markers like CD44-FITC (99.7%), CD73-PE (99.8%), CD90-PerCP (99.7%), CD29-PE (99.9%), and CD34-APC (0.102%) (Fig. S1). HEK293 and 293 T cell lines were cultured with DMEM medium (Gibco, Grand Island, NT, USA) with 10% FBS. The proliferation medium (PM) was DME/F12 medium with 10% FBS. The osteogenic differentiation medium (OM) was DME/F12 medium with 10% FBS supplemented with the following differentiation compositions: 50 mg/L ascorbic acid, 10 mM β-glycerophosphate, and 10^−7^ M dexamethasone (Sigma, St. Louis, MO, USA). hUC-MSCs were induced for osteogenic differentiation in OM for 7, 14, 21, or 28 days for further experiments.

### Energy dispersive spectroscopy (EDS)

The samples for scanning electron microscopy (SEM) and energy dispersive spectroscopy (EDS) analyses were mounted on aluminum sample holders using double-sided and electrically conductive adhesive tape, followed by sputtering with gold for 30 s using a sputter coater (LJ-16, Beijing Yulong Times Technology Co., China) to increase their electrical conductivity^52,53^. Then, the microstructure and elemental composition of the samples were characterized by a Scanning Election Microscope (PhenomProX, Phenom-World BV, Netherlands) equipped with an EDS (INCA, Oxford Instruments, UK) under an acceleration voltage of 15 kV.

### Microarray and bioinformatic analysis

After 21 days of culture in PM or OM, hUC-MSCs were harvested and the lncRNA expression profile was obtained from OE Biotech (Shanghai, China) using Affymetrix WT (Thermo Fisher Scientific, Waltham, MA, USA). For the filtering of differentially expressed lncRNAs, fold change values < −2.0 and >2.0 and *p*-values at <0.05 were used for the analysis.

### Plasmid construction

Full-length ODIR1 cDNA was amplified from hUC-MSCs and cloned into pCDH vector (pCDH-ODIR1) for stable or transient expression of ODIR1, and the Full-length ODIR1 was also cloned into the pcDNA3.1/His C vector (pcDNA3.1-ODIR1) for luciferase and RNA pull down assays. The shODIR1 sequences were synthetized by BBI Life Science (Shanghai, China) and subcloned into the pLVTH vector (pLVTH-shODIR1) for stable knockdown of ODIR1. The OSX and RUNX2 promoter regions were amplified from hUC-MSCs cDNA and subcloned into the pMIR vector (pMIR-OSX-Luc and pMIR-RUNX2-Luc) for the luciferase assay. All plasmids were maintained in DH5α competent cells, and plasmid DNA was extracted using Plasmid Mini Kit I (OMEGA, Norcross, USA) following the manufacturer’s instructions.

### Cell transfection

Target siRNAs (siODIR1, si-OSX, si-RUNX2, siBARD1, siCUL3, and siFBXO25) or negative control siRNA were obtained from GenePharma (Shanghai, China). SiRNA transfections were performed using 100 nM siRNA and Lipofectamine 2000 Reagent (Invitrogen, Waltham, MA, USA) following the manufacturer’s instructions. Plasmid transfections were performed using 2 μg plasmid and Lipofectamine 2000 Reagent in 6-well plates according to the manufacturer’s instructions. Total RNA and protein were extracted from hUC-MSCs after transfection for 24 and 48 h, respectively.

### Stable cell line establishment

The pCDH-ODIR1 (10 μg) or pLVTH-shODIR1 (μg) and corresponding control vector were co-transfected with two packaging vectors (7.5 μg pSPAX2 and 7.5 μg pMD2G) into 293FT cells for 48 h to produce lentivirus. The supernatant medium containing target lentivirus was collected and filtrated by 0.22 μM aperture PES membranes (Millipore, Darmstadt, Germany). Then hUC-MSCs were infected with lentivirus mixed with 1:1000 Polybrene (Santa Cruz Biotechnology, CA, USA). The infection efficiency was detected by BX60 Fluorescence Microscope (Olympus, Japan) after 48 h of infection. The GFP positive cells were analyzed and sorted by Flow Cytometry and subsequently cultured for further experiments.

### Real-time quantitative PCR (RT-qPCR)

Total RNA was extracted from QC1205 and 293FT cells using TRIzol reagent (Life Technologies, Carlsbad, CA, USA). Then reverse transcription (RT) of total RNA was conducted by RevertAid First Strand cDNA Synthesis Kit (Thermo Fisher Scientific, Waltham, MA, USA) using random primers. RT-qPCR was performed using 2× SYBR Green qPCR Master Mix (Biotool, HOU, USA) and a program conducted on Real-Time PCR CFX96 Instrument (Bio-Rad, Hercules, CA, USA). GAPDH and β-Actin were used as internal references for analyzing relative RNA levels. Relative RNA levels were obtained from the Bio-Rad CFX manager (Bio-Rad, Hercules, CA, USA). The primers are listed in Table S4, and all samples were performed in triplicate independent tests.

### Western blotting

Cells were harvested and then lysed with RIPA lysis buffer containing 1.0 mM protease inhibitor cocktail, 1.0 nM DTT and PMSF for 30 min at 4 °C. Then, the protein supernatant was collected using a microcentrifuge at 13,000 rpm for 15 min at 4 °C. Appropriate protein (50 μg) of samples were subjected to 10–12% SDS-PAGE gel electrophoresis and electroblotted into PVDF membranes (Millipore, Darmstadt, Germany) as previously described^54^. The membranes were blocked with skimmed milk solution (5%) and incubated with the following primary antibodies: GAPDH (1:3000; Origene, USA), β-Actin (1:3000; Abclonal, USA), Osteocalcin (1:1000; Santa Cruz Biotechnology, USA), RUNX2 (1:1000; Proteintech, China), SP7/Osterix (1:1000; Abcam, USA), BARD1 (1:500; Santa Cruz Biotechnology, USA), FBXO25 (1:300; Santa Cruz Biotechnology, USA), CUL3 (1:500; Santa Cruz Biotechnology, USA), H2A (1:1000; Absin, China), H2B (1:1000; Abcam, USA), H3 (1:1000; CST, USA), H2AK119ub (1:1000; CST, USA), H2BK120ub (1:1000; CST, USA), and H3K4me3 (1:2000; CST, USA). The results were visualized using a MiniChemi 610 System Instrument (Beijing, China).

### In vitro ubiquitination assay

To determine whether ODIR1 regulates the protein expression of FBXO25 at the post-transcriptional level, the proteasome inhibitor MG132 and the protein synthesis inhibitor cycloheximide (CHX; Sigma Aldrich, Shanghai, China) were applied to evaluate the protein stability or synthesis ability of FBXO25, respectively. hUC-MSCs transfected with negative vector or ODIR1 full length expression vector were cultured in PM containing MG132 (100 ng/ml) or cycloheximide (CHX) (100 μg/ml) for 8 h, respectively. The hUC-MSCs with sh-NC and sh-ODIR1 were incubated with PM containing MG132 (100 ng/ml) or cycloheximide (CHX) (100 μg/ml) for 8 h, respectively. After treatment, the total protein was extracted for western blotting analysis. For the in vitro ubiquitination assays, HEK293T cells were transfected with GFP-FBXO25, Myc-H2B and HA-Ub or ODIR1 as indicated. After 48 h of culture, the levels of ubiquitinated H2B were monitored by IP analysis, followed by western blotting with the indicated antibodies.

### Immunofluorescence and in situ hybridization

A total of 5.0 × 10^3^ hUC-MSCs cells were seeded in 6-well plates for 24 h. Then, the cells were fixed with 4% PFA (paraformaldehyde; Bioshrap, China) solution and the following steps were monitored as previously described^55,56^. The images were visualized and collected using UltraVIEW VoX (PerkinElmer, Waltham, USA) confocal fluorescence microscope. In situ hybridization was carried out following the manufacturer’s protocol. The synthetic ODIR1 Digoxin tag probe sequences (Table S4) were obtained from Sangon Biotech (Shanghai, China).

### Alizarin red S (ARS) staining and ALP staining

The hUC-MSCs were seeded in 6-well plates. Osteogenic induction was performed in OM when the cells reached 60–80% confluence, and the negative control cells were cultured in PM. After 21 days of osteogenic differentiation, cells were fixed with 4% PFA solution and ARS staining was performed using the ARS Kit (Sigma, USA) following the manufacturer’s instructions. After osteogenic differentiation for 14 days, cells were fixed with 4% PFA solution and ALP staining was performed using the ALP Kit (Beyotime, Shanghai, China) and following the manufacturer’ s instructions.

### RNA pull-down assay

Full length ODIR1 RNA (ODIR1) and ODIR1-antisense RNA (ODIR1-AS) were transcribed in vitro from pcDNA3.1-ODIR1 and pcDNA3.1-ODIR1-AS and labeled with biotin by the Biotin RNA Labeling Mix (Sigma, USA) and T7 RNA polymerase (Sigma, USA). The biotin-labeled RNAs (3 μg) was subjected to heat shock at 90 °C for 2 min and cooled at room temperature (about 25.0 °C) for 20 min, folded with RNA structure buffer, then mixed with cell extract of hUC-MSCs, incubated with Streptavidin Agarose Resin (Thermo Fisher Scientific Inc., Waltham, MA, USA) at 4 °C for 2 h and washed. The retrieved proteins were subjected to SDS-PAGE gel electrophoresis and the protein band of the ODIR1 group was subjected to silver staining, and differential protein bands were identified using high resolution mass spectrometry (LC-LTQ-Orbitrap; Thermo Fisher Scientific, Waltham, MA, USA). The identified proteins were examined using regular western blotting assay. The GO functional annotation was analyzed using the Gene Ontology Consortium (http://www.geneontology.org/).

### RNA immunoprecipitation (RIP)

The cells extract of hUC-MSCs was admixed with Protein-G/A Plus Agarose (Santa Cruz Biotechnology, USA), and then incubated with 2 μg antibodies against FBXO25, CUL3, BARD1 or normal rabbit anti-IgG at 4 °C for 4 h and washed. Then, the samples were subjected to RT-qPCR. The fold enrichment of precipitated ODIR1 RNA was examined by RT-qPCR. The primers used are listed in Table S4. All samples were detected in triplicate.

### ChIP

The hUC-MSCs were induced with OM for 7 days or were transfected with siODIR1 for 48 h. Then the cells were cross-linked with 1% PFA solution for 10 min at 37 °C and the cross-linked media were neutralized with 0.125 M Glycine (Bioshrap, China) for 10 min at room temperature (~25 °C). Then, cells were harvested and lysed by SDS lysis buffer at 4 °C with rotation vortexing for 30 min, and the supernatant lysate was sonicated using Cole-Parmer Instruments CP130 (Vernon, Illinois, USA) for 15 min at the frequency of 1.0 s ultrasound and 1.0 s stop (for one cycle). After centrifugation at 13,000 rpm for 10 min at 4.0 °C, the supernatant was collected and mixed with the antibodies against H2AK119ub, H2BK120ub, and H3K4me3 (2 μg each antibody), incubated with Protein-A/G Immunoprecipitation Magnetic Beads (Santa Cruz Biotechnology, USA) for 2 h at 4.0 °C and washed using wash buffer. The immunoprecipitated DNAs were de-cross-linked using 10% Chelex-100 mixture (Bio-Rad, Hercules, CA, USA) for 10 min at 99.0 °C, and the DNA supernatant was collected by centrifugation at 13,000 rpm for 3 min at 4 °C. The immunoprecipitated DNAs were detected by RT-qPCR using the OSX and RUNX2 promoter primers (Table S4). The relative fold enrichment of H2AK119ub, H2BK120ub and H3K4me3 at the OSX or RUNX2 promoter region was to the Input and the immunoprecipitation values were normalized to normal rabbit IgG.

### Luciferase assay

HEK293T cells were collected and seeded at 1.0 × 10^5^ cells in 24-well plates per well, and then pcDNA3.1-ODIR1 was co-transfected with pMIR-OSX-Luc or pMIR-RUNX2-Luc when the cells grow to 80% confluence. For further verification, the pcDNA3.1-ODIR1 and pcDNA3.1-NC, siODIR1 and siNC plasmids were co-transfected with pMIR-OSX-Luc into 293T cells. For ODIR1 degree dependent luciferase assay, different amounts (0.1, 0.2, 0.5, and 1.0 μg) of pcDNA3.1-ODIR1 were co-transfected with pMIR-OSX-Luc into 293 T cells. The cells of each well were co-transfected with pRL-TK (Renilla), which was used as internal reference. The dual-luciferase assays were monitored using the Dual-Luciferase Reporter Assay System (Promega, WI, USA) following the manufacturer’s instructions. All samples were detected in triplicate independent tests.

### Bone formation in vivo

The stable overexpression of ODIR1 in hUC-MSCs was induced in OM at 37 °C for 7 days. Then the cells were harvested and placed on the surface of Bio-Oss Collagen scaffolds (5 × 5 × 1.75 mm^3^) (Geistlich; Pharma, Australia) at 37 °C for 1 h, and the incubated scaffolds were injected subcutaneously in either back of BALB/c nude mice (5 weeks old, *n* = 4 per group). This experiment was approved by the Institutional Animal Care and Use Committee of Peking University Health Science Center (LA2014233). Animal experiments were carried out according to the Guidelines of Department of Laboratory Animals (Central South University, China).

### In vivo bone formation analyses

After 8 weeks of osteogenic differentiation in vivo, all nude mice were sacrificed, and the Bio-Oss Collagen was stripped from the subcutaneous skin and fixed in 4% PFA. The specimens were analyzed using Quantum GX microCT Imaging System (PerkinElmer, CA, USA). BV/TV and BMD were measured using Quantum Supporting Xcapture Software (PerkinElmer, CA, USA). Then the specimens were decalcified in 10% EDTA solution for 14 days, followed by embedding with paraffin and sectioning. The sections (4.0 μm) were stained with H and E (Boster, USA) and Masson Trichrome (Solarbio, Beijing, China). In addition, IHC staining was performed with anti-OCN and anti-OSX to evaluate the expression of osteogenic markers. The images were taken using CKX41 Optical microscope (Olympus, Japan).

### Statistical analysis

All data are presented as Mean ± SD of three independent experiments and statistical analysis was performed using SPSS Statistics V22.0 (IBM Corporation, Armonk, NY, USA). Two-group data were analyzed using Student’s *t*-test, and three-group or multiple-group data were analyzed using one-way ANOVA. A two-tailed *p*-value < 0.05 was regarded as statistically significant.

## Supplementary information

Supplemental Figure 1

Supplemental Figure 2

Supplemental Figure 3

Supplemental Figure 4

Supplemental Figure 5

Supplemental Figure 6

Supplemental Figure Legends

Table S1

Table S2

Table S3

Table S4
