# Supplementary figures and images for: LncRNA ODIR1 inhibits osteogenic differentiation of hUC-MSCs through the FBXO25/H2BK120ub/H3K4me3/OSX axis
Source: Cell Death Dis. 2019 Dec 11;10(12):947. doi: 10.1038/s41419-019-2148-2 (PMC6906393; doi:10.1038/s41419-019-2148-2)

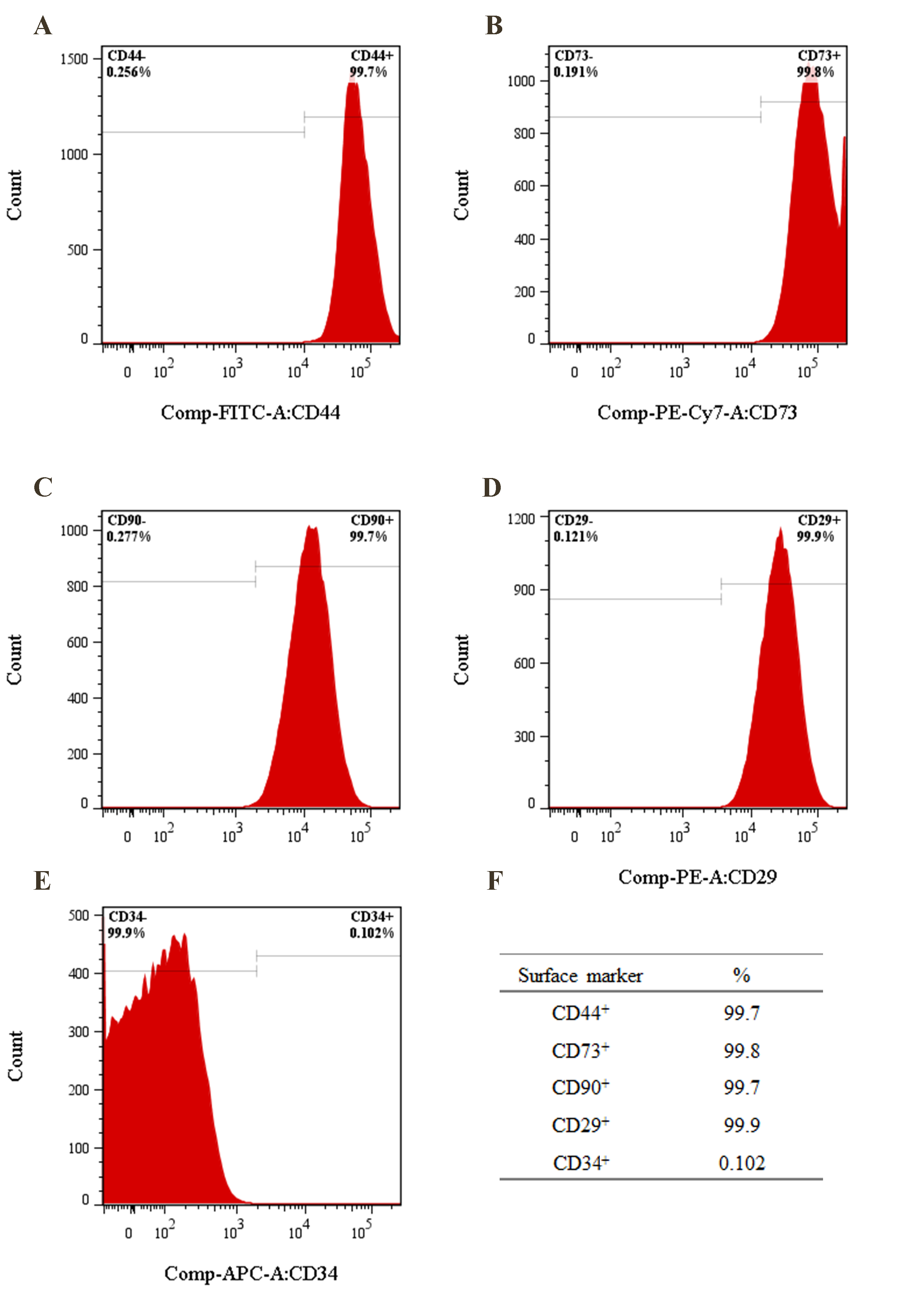

Supplement: Supplementary file 1 — Supplemental Figure 1 [file 41419_2019_2148_MOESM1_ESM.tif]

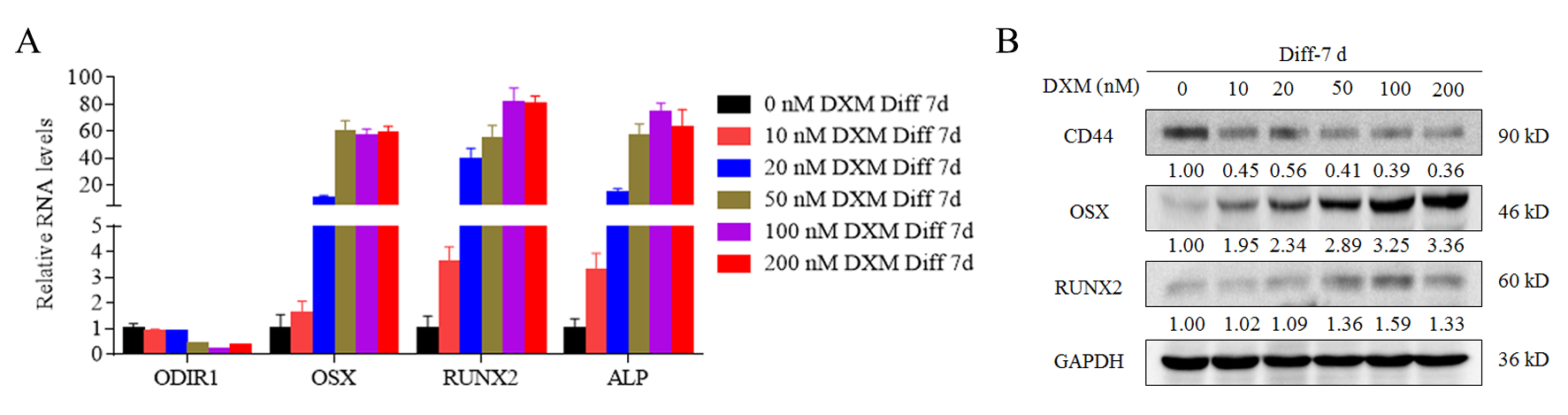

Supplement: Supplementary file 2 — Supplemental Figure 2 [file 41419_2019_2148_MOESM2_ESM.tif]

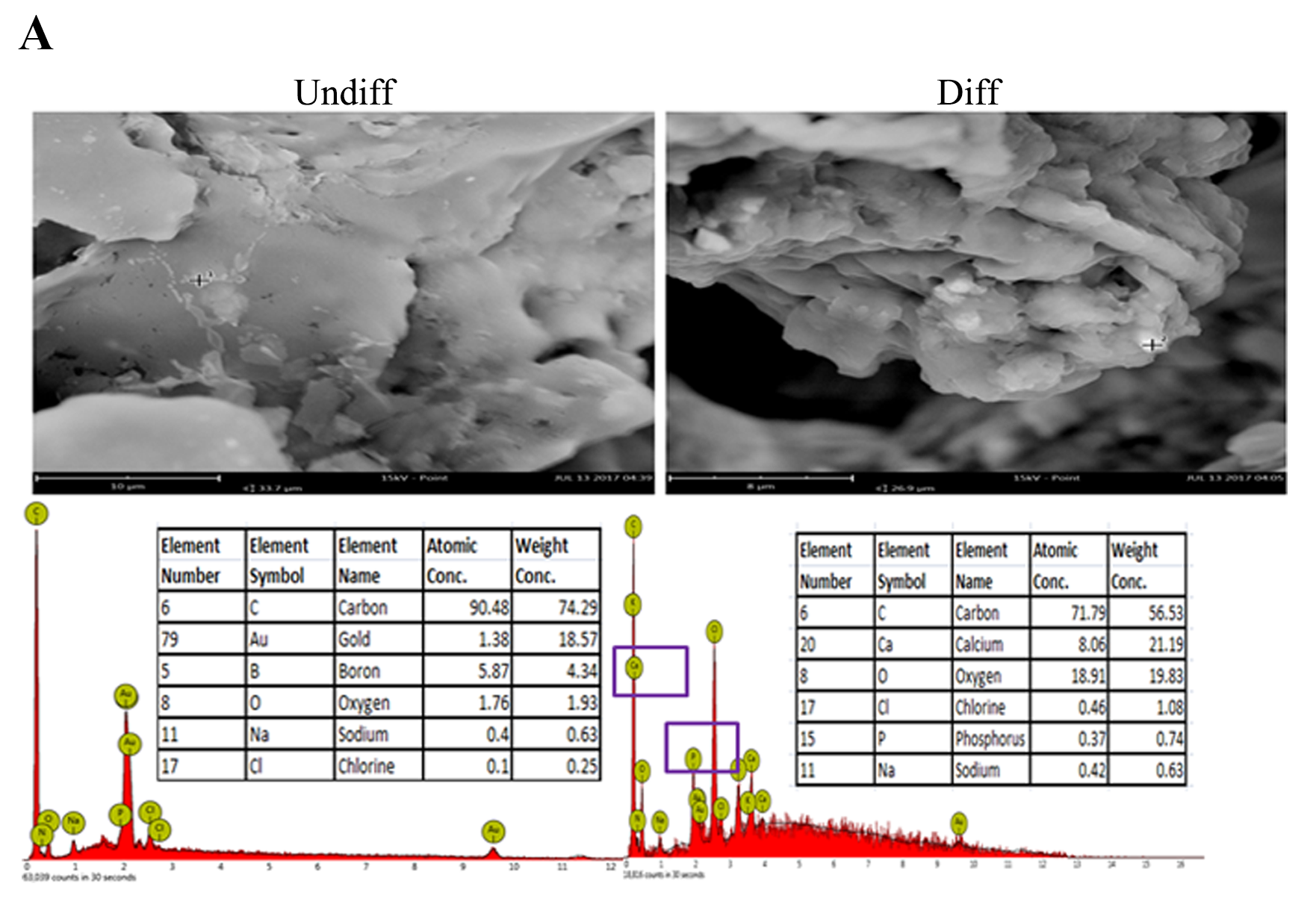

Supplement: Supplementary file 3 — Supplemental Figure 3 [file 41419_2019_2148_MOESM3_ESM.tif]

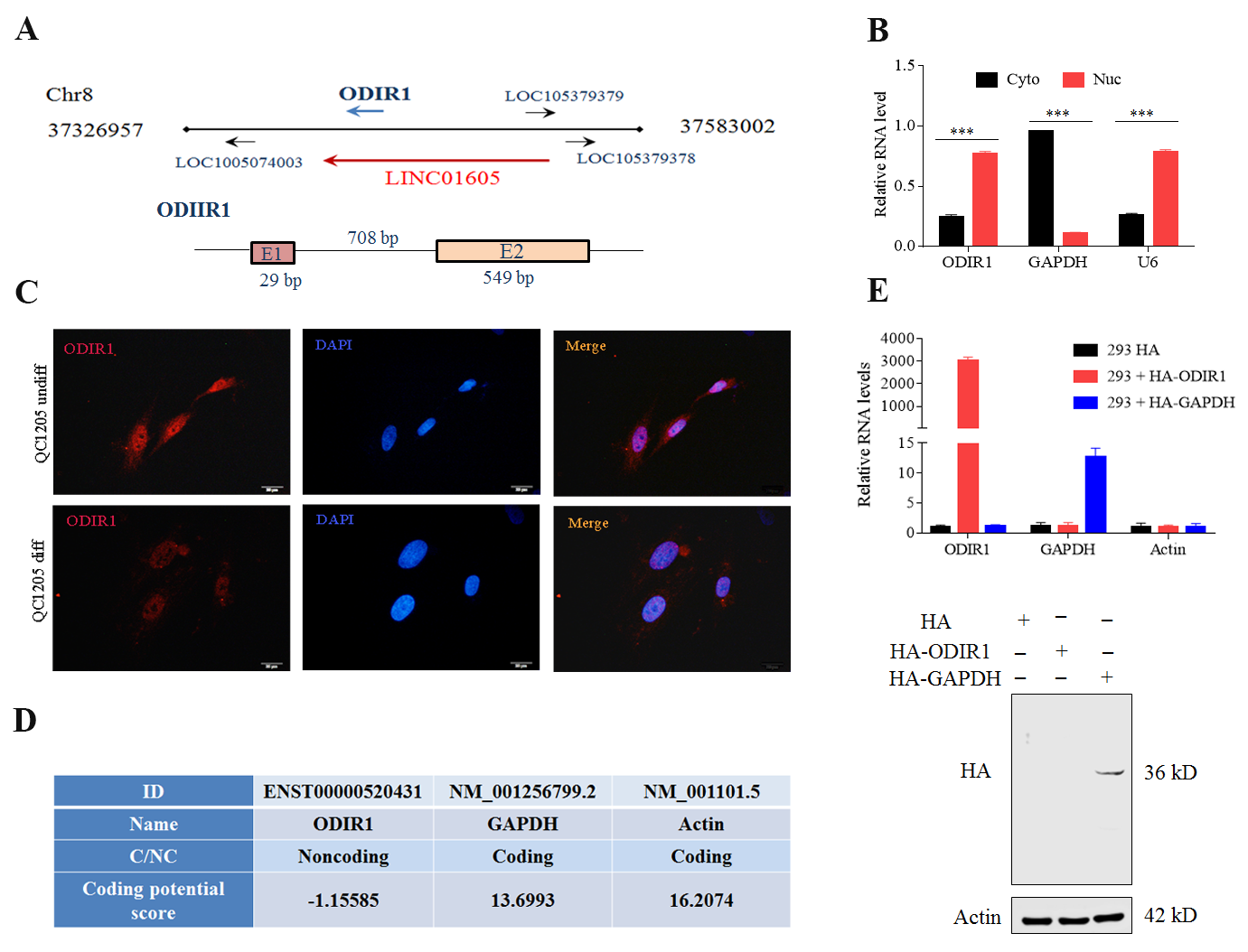

Supplement: Supplementary file 4 — Supplemental Figure 4 [file 41419_2019_2148_MOESM4_ESM.tif]

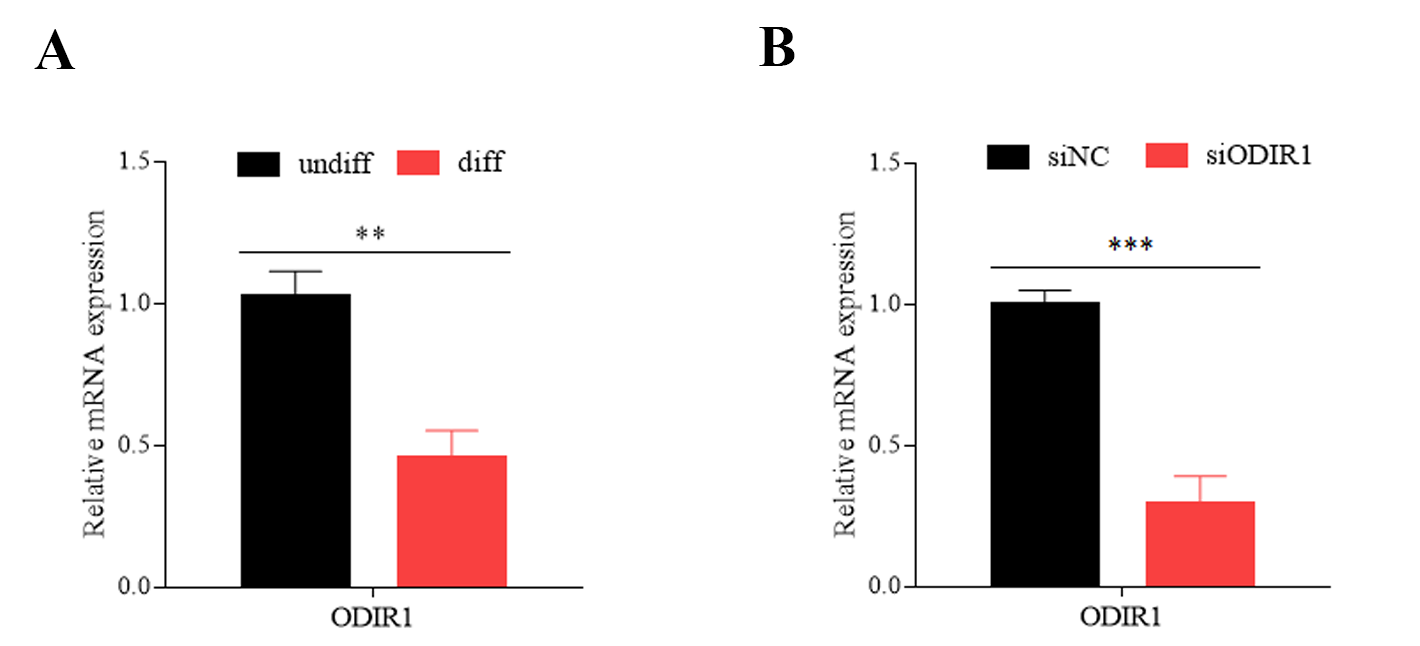

Supplement: Supplementary file 5 — Supplemental Figure 5 [file 41419_2019_2148_MOESM5_ESM.tif]

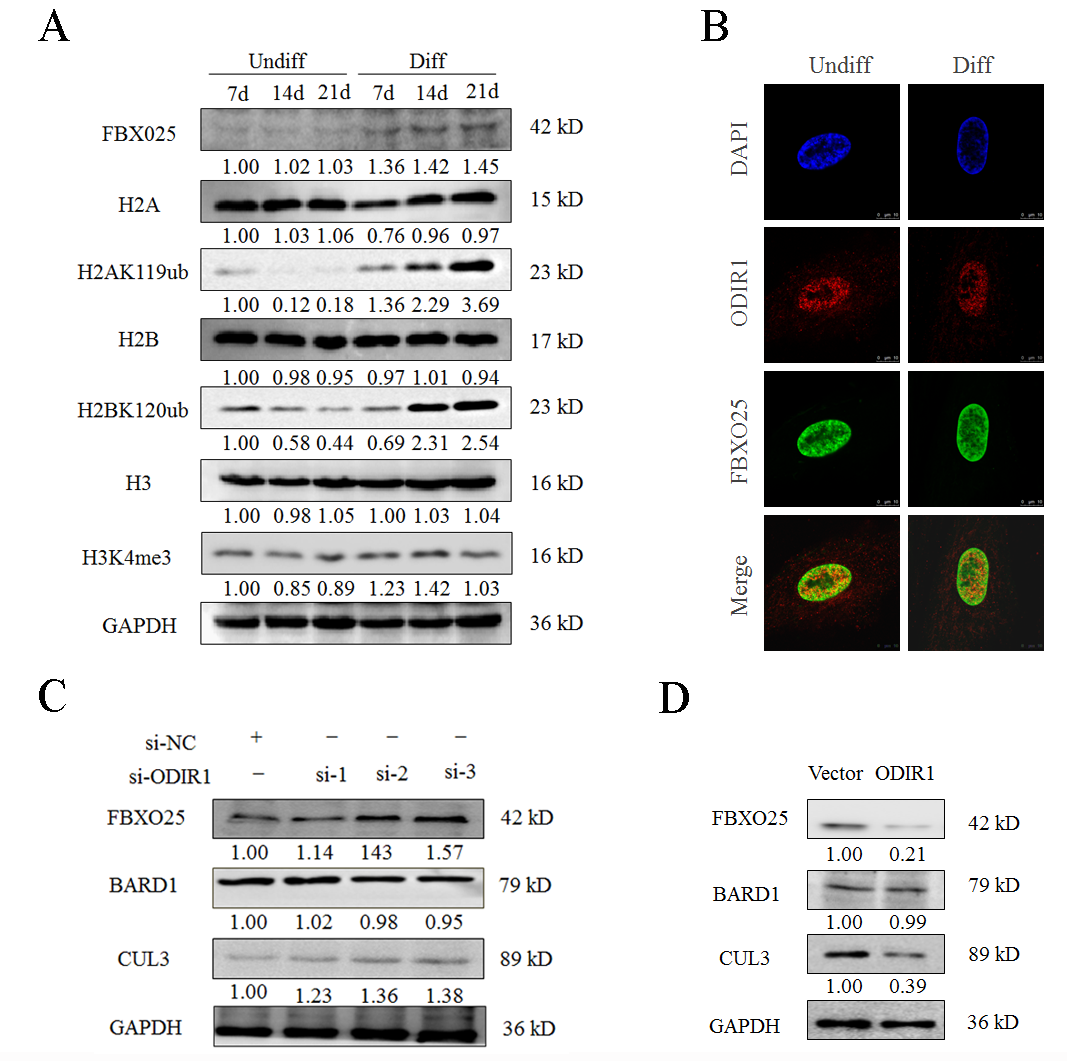

Supplement: Supplementary file 6 — Supplemental Figure 6 [file 41419_2019_2148_MOESM6_ESM.tif]
