# Supplementary material for: LncRNA ODIR1 inhibits osteogenic differentiation of hUC-MSCs through the FBXO25/H2BK120ub/H3K4me3/OSX axis
Source: Cell Death Dis. 2019 Dec 11;10(12):947. doi: 10.1038/s41419-019-2148-2 (PMC6906393; doi:10.1038/s41419-019-2148-2)
Supplement: Supplementary file 7 — Supplemental Figure Legends [file 41419_2019_2148_MOESM7_ESM.docx]

**Supplemental Figure Legends**

**Supplemental Figure 1 The QC1205 cell line was characterized using Flow Cytometry for MSCs specific surface markers.**

(A-E) MSCs specific surface markers, including CD44, CD73, CD90, CD29 and CD34, were detected using Flow Cytometry. (F) The percentage of surface markers positive or negative hUC-MSCs were counted using Flow Cytometry.

**Supplemental Figure 2 The optimum concentration of dexamethasone (DMX) were selected for hUC-MSCs differentiation**

(A) The hUC-MSCs were incubated with osteogenic differentiation medium containing different concentrations of DMX and induced to differentiate to osteoblast for 7 days, the RNA levels of ODIR1, OSX, RUNX2 and ALP were determined by RT-qPCR, and (B) the proteins levels of CD44, OSX and RUNX2 were analyzed by western blotting.

**Supplemental Figure 3 Elements analysis of calcium nodules derived form hUC-MSCs differentiation**

The hUC-MSCs were induced in osteogenic differentiation medium (OM) or proliferation medium (PM) for 28 days and analyzed by scanning electron microscopy for elements. The element calcium (Ca) and phosphorus (P) were detected in differentiated group. Scale bar, 20 μm. Ca and P elements can be detected in osteogenic differentiated calcium nodules.

**Supplemental Figure 4 ODIR1 is verified as a non-protein coding RNA.**

(A) ODIR1 locates in chromosome 8 and is the (-) DNA strand transcription. ODIR1 contains two exons, 29 and 549 bp, respectively. (B) ODIR1 mainly located at cellular nucleus of hUC-MSCs. (C) The expression and location of ODIR1 in hUC-MSCs was detected by *in situ* hybridization assay. (D) The coding potential of ODIR1, GAPDH and Actin sequences were analyzed by Coding Potential Calculator (CPC) program. (E) Relative RNA levels of ODIR1 and GAPDH were monitored by RT-qPCR assay and relative proteins levels of ODIR1 and GAPDH were analyzed by western blotting in 293T cells after transfected with a control vector, HA-ODIR1 or HA-GAPDH, respectively. ODIR1 was predicted to be a non-protein coding RNA.

**Supplemental Figure 5 RNA levels of ODIR1 in hUC-MSCs for ChIP assay.**

(A) The hUC-MSCs were induced to differentiate to osteoblast in OM or PM for 7 days, and the RNA levels of ODIR1 was measured by RT-qPCR in hUC-MSCs lysates. (B) The hUC-MSCs were transfected with NC or ODIR1 siRNA, and the RNA levels of ODIR1 were measured by RT-qPCR in hUC-MSCs lysates.

**Supplemental Figure 6 The expression change of ODIR1 pull-down proteins during hUC-MSCs osteogenic differentiation.**

(A) The hUC-MSCs were induced to differentiate to osteoblast in OM or PM for 7, 14 and 21 days, respectively, and the protein levels of FBXO25 and histones marks were measured by western blotting in hUC-MSCs lysates. (B) FISH assay detects the co-localization of ODIR1 and FBXO25 in in hUC-MSCs. (C) The hUC-MSCs were transfected with siNC and ODIR1 siRNAs, the proteins levels of FBXO25, BARD1 and CUL3 were analyzed by western blotting assay. (D) The hUC-MSCs were transfected with vector and ODIR plasmid, the proteins levels of FBXO25, BARD1 and CUL3 were analyzed by western blotting assay.
