# Supplementary material for: LncRNA ODIR1 inhibits osteogenic differentiation of hUC-MSCs through the FBXO25/H2BK120ub/H3K4me3/OSX axis
Source: Cell Death Dis. 2019 Dec 11;10(12):947. doi: 10.1038/s41419-019-2148-2 (PMC6906393; doi:10.1038/s41419-019-2148-2)
Supplement: Supplementary file 8 — Table S1 [file 41419_2019_2148_MOESM8_ESM.docx]

**Table 1 Differentially expressed lncRNAs of osteogenic differentiated hUC-MSCs compared to undifferentiated hUC-MSCs**

| LncRNAs | Log_2_(Fold change) | Undiff | Diff | *p*-value |
| --- | --- | --- | --- | --- |
| **Up-regulated** | | | | |
| LOC100506530 | 4.743904391 | 0.479845 | 12.857558 | 5.73E-47 |
| ENST00000429456 | 3.203696958 | 1.166168 | 10.744099 | 2.89E-47 |
| ENST00000511346 | 2.75079508 | 1.659496 | 11.169868 | 9.95E-26 |
| ENST00000430247 | 2.179378768 | 2.231545 | 10.107972 | 3.63E-53 |
| ENST00000423708 | 2.130618166 | 2.264958 | 9.918366 | 6.06E-52 |
| ENST00000499173 | 2.0936245 | 1.659896 | 7.084752 | 4.28E-42 |
| ENST00000442389 | 1.991539042 | 1.032659 | 4.106482 | 8.72E-36 |
| ENST00000428508 | 1.779594658 | 0.994985 | 3.416079 | 6.49E-34 |
| ENST00000430555 | 1.690403883 | 0.949486 | 3.064438 | 1.84E-34 |
| ENST00000555403 | 1.618146955 | 2.065959 | 6.342091 | 9.64E-39 |
| ENST00000519603 | 1.447858217 | 1.279762 | 3.491226 | 2.49E-21 |
| ENST00000515444 | 1.319286881 | 1.285896 | 3.208860 | 9.89E-28 |
| ENST00000499583 | 1.147862878 | 1.689887 | 3.744543 | 3.99E-23 |
| ENST00000550678 | 1.130663106 | 1.265987 | 2.771997 | 1.21E-32 |
| ENST00000433303 | 1.072665353 | 2.065980 | 4.345408 | 1.53E-25 |
| ENST00000529841 | 1.034941977 | 2.032949 | 4.165576 | 4.87E-18 |
| **Down-regulated** | | | | |
| ENST00000448580 | -1.238472 | 3.359485 | 1.423821 | 7.06E-27 |
| ENST00000554254 | -1.801152446 | 3.984985 | 1.143473 | 2.68E-20 |
| ENST00000505289 | -1.867799227 | 3.949754 | 1.082197 | 6.23E-23 |
| ENST00000546273 | -2.092734142 | 3.265557 | 0.765564 | 1.60E-25 |
| ENST00000511520 | -2.397739449 | 7.269768 | 1.379523 | 4.57E-27 |
| ENST00000520431 | -2.555109539 | 6.659947 | 1.133199 | 3.87E-28 |
| ENST00000539116 | -2.644905398 | 5.254547 | 0.840116 | 2.17E-22 |
