# Supplementary material for: LncRNA ODIR1 inhibits osteogenic differentiation of hUC-MSCs through the FBXO25/H2BK120ub/H3K4me3/OSX axis
Source: Cell Death Dis. 2019 Dec 11;10(12):947. doi: 10.1038/s41419-019-2148-2 (PMC6906393; doi:10.1038/s41419-019-2148-2)
Supplement: Supplementary file 9 — Table S2 [file 41419_2019_2148_MOESM9_ESM.docx]

**Table 2 Proteins of ODIR1 pull-down product were analyzed by mass spectrometry**

| **Description** | **Gene Name** | **Score** | **MW [kDa]** |
| --- | --- | --- | --- |
| Trypsin-1 | PRSS1 | 39.12 | 15.4 |
| ELAV-like protein 1 | ELAVL1 | 36.72 | 36.1 |
| Heterogeneous nuclear ribonucleoprotein A1 | HNRNPA1 | 29.36 | 33.1 |
| Heterogeneous nuclear ribonucleoprotein H | HNRNPH1 | 25.17 | 47.1 |
| BRCA1-associated RING domain protein 1 | BARD1 | 24.66 | 24.4 |
| Albumin, isoform CRA_k | ALB | 21.98 | 47.3 |
| Alpha-2-macroglobulin | A2M | 21.33 | 163.2 |
| F-box only protein 25 | FBXO25 | 21.30 | 43.3 |
| Serum albumin | ALB | 20.60 | 45.1 |
| Cullin-3 | CUL3 | 20.44 | 39.1 |
| Myosin-9 | MYH9 | 20.12 | 226.4 |
| Inactive caspase-12 | H3 | 19.14 | 38.8 |
| Histone H2B type 1-K | HIST1H2BK | 18.54 | 13.9 |
| Histone H2A type 1-H | HIST1H2AH | 16.40 | 13.9 |
| Apolipoprotein A-I | APOA1 | 15.45 | 27.9 |
| Leucine-rich repeat neuronal protein 4 | LRRN4 | 13.76 | 78.8 |
| Histone H4 | HIST1H4A | 12.66 | 11.4 |
| Heterogeneous nuclear ribonucleoprotein D-like | HNRNPDL | 10.68 | 40.0 |
| Heterogeneous nuclear ribonucleoprotein A/B | HNRNPAB | 9.31 | 30.3 |
| Lupus La protein | SSB | 8.96 | 46.8 |
| Annexin | ANXA1 | 8.52 | 22.7 |
| Lactotransferrin | LTF | 7.38 | 76.6 |
| Phospholipid-transporting ATPase IC | ATP8B1 | 6.82 | 143.6 |
| Annexin A2 | ANXA2 | 6.58 | 5.3 |
| Fatty acid-binding protein, heart | FABP3 | 5.84 | 10.2 |
| Serotransferrin | TF | 4.04 | 14.7 |
| Propionyl-CoA carboxylase beta chain | PCCB | 3.53 | 13.9 |
| Alpha-2-HS-glycoprotein | AHSG | 3.28 | 39.3 |
| Protein Hook homolog 1 | HOOK1 | 3.24 | 84.6 |
| ATP-binding cassette sub-family A member 7 | ABCA7 | 3.09 | 64.6 |
| ATP synthase subunit alpha | ATP5A1 | 3.03 | 8.3 |
| 60 kDa heat shock protein | HSPD1 | 2.72 | 23.8 |
| Desmoplakin | DSP | 2.65 | 331.6 |
| Annexin A6 | ANXA6 | 2.55 | 75.8 |
| Zinc finger protein 518A | ZNF518A | 2.45 | 166.7 |
| Intersectin-1 | ITSN1 | 2.33 | 36.3 |
| Crescerin-1 | FAM179B | 2.28 | 189.2 |
| SOSS complex subunit B2 | NABP1 | 2.19 | 22.4 |
| Nucleolar protein 56 | NOP56 | 2.17 | 24.2 |
