# Supplementary material for: LncRNA ODIR1 inhibits osteogenic differentiation of hUC-MSCs through the FBXO25/H2BK120ub/H3K4me3/OSX axis
Source: Cell Death Dis. 2019 Dec 11;10(12):947. doi: 10.1038/s41419-019-2148-2 (PMC6906393; doi:10.1038/s41419-019-2148-2)
Supplement: Supplementary file 10 — Table S3 [file 41419_2019_2148_MOESM10_ESM.docx]

**Table 3 GO annotations for ODIR1 pull-down proteins**
