# Supplementary material for: LncRNA ODIR1 inhibits osteogenic differentiation of hUC-MSCs through the FBXO25/H2BK120ub/H3K4me3/OSX axis
Source: Cell Death Dis. 2019 Dec 11;10(12):947. doi: 10.1038/s41419-019-2148-2 (PMC6906393; doi:10.1038/s41419-019-2148-2)
Supplement: Supplementary file 11 — Table S4 [file 41419_2019_2148_MOESM11_ESM.docx]

**Table 4 Primers and constructs sequences**

| **Name** | **Forward Primer (5'-3')** | **Reverse Primer (5'-3')** |
| --- | --- | --- |
| **Primers for ODIR1 sense strand and antisense strand** | | |
| ODIR1 sense | GCTGGCTAGCGTTTAAACTTAAGCTTAAAGTGCTTTGAACAGCGTTTG | TGCAGAATTCCACCACACTGGATCCCTCTGTCGTCACCTTGATCTTGG |
| ODIR1 antisense | GCTGGCTAGCGTTTAAACTTGGATCCAAAGTGCTTTGAACAGCGTTTG | TGCAGAATTCCACCACACTAAGCTTCTCTGTCGTCACCTTGATCTTGG |
| **Primers for shODIR1 construct** | | |
| shODIR1-1  shODIR1-2 | GATCTGCAAACGGAAGAGCAGCAATTCAAGAGATTGCTGCTCTTCCGTTTGCTTTTTA  GATCTGCATAGAGATGGCCTTCTTTTCAAGAGAAAGAAGGCCATCTCTATGCTTTTTA | AGCTTAAAAAGCAAACGGAAGAGCAGCAATCTCTTGAATTGCTGCTCTTCCGTTTGCA  AGCTTAAAAAGCATAGAGATGGCCTTCTTTCTCTTGAAAAGAAGGCCATCTCTATGCA |
| **Primers for OSX and RUNX2 promoter region constructs** | | |
| OSX promoter | ATATTACGCGTCACACACGGGCACGTACA | GCGCAAGCTTTTAAGGTTGTGGCTGGTT |
| RUNX2 promoter | GCGCGACTAGTTTTCCCAAAGATGTTTCC | ATATTGCCGGCAGCACTCACTGACTCTG |
| **Primers for qPCR** | | |
| ODIR1 | GGCACATGGAGAATTAAATG | TCTTGTCCTCGCCTTGTCTT |
| LOC100506530 | ATGCAATGTTTCGATCATGG | ACCATCCCCAGGGTCTTAGT |
| ENST00000429456 | TGAAGGGAGGAAGATGGTGT | GAAGGTGAAGGAGGAGCAGA |
| ENST00000430247 | GCATTGGGATTACAGGGAGA | CTTTTCACCAGCCTCTCCAG |
| ENST00000511346 | CTTCAGTCTCCCAGGATCGT | TCGAGAAACCGAACACTGATT |
| ENST00000529841 | ACTACTTGGCCTGCTCAGTG | GCATAGCAAAACCCCATCTC |
| OPN | CATCACCTGTGCCATACCAG | GTCATGGCTTTCGTTGGACT |
| OCN | GACTGTGACGAGTTGGCTGA | CTGGAGAGGAGAAGAACTGG |
| CD44 | CACAACAACACAAATGGCTG | CAATGCCTGATCCAGAAAAA |
| RUNX2 | GAGTGGACGAGGCAAGAGTT | GAGGCGGTCAGAGAACAAAC |
| OSX | CTTGTGCCTGATACCTGCACT | TCACTCTACCTGACCCGTCATC |
| Col1a1 | AGCCAGCAGATCGAGAACAT | TCTTGTCCTTGGGGTTCTTG |
| ZNF518A | CACCGGCAGTAAGATCAGCA | TCCCAGGACCTCTTTCTTCA |
| CUL3 | GACGAGCACCATGTCGAATC | ATCCATGGTCATCGGAAAGGC |
| FBXO25 | TGCCATTTTTGGGTCAGGACT | GCCATGCCTTTTGAGTATTTGTG |
| BARD1 | AGGAGCCTTTCATCCGAAGG | TCAGAATGTTAGTACAACGCGA |
| ALP | CTCCAACATGGACATTGACG | GGCTCAAAGAGACCCATGAG |
| Actin | TCACCAACTGGGACGACATG | GTCACCGGAGTCCATCCGAT |
| GAPDH | AACGGATTTGGTCGTATTGG | TTGATTTTGGAGGGATCTCG |
| Tubulin | CGTGTTCGGCCAGAGTGGTGC | GGGTGAGGGCATGACGCTGAA |
| U6 | CTCGCTTCGGCAGCACA | AACGCTTCACGAATTTGCGT |
| **Primers for RIP** | | |
| ODIR1-rip | GGCACATGGAGAATTAAATG | TCTTGTCCTCGCCTTGTCTT |
| GAPDH-rip | tcttgactcaccctgccct | acaaaggcactcctggaaac |
| **Primers for ChIP** | | |
| OSX P1 | GAAGCTCTGACAACTTGCCC | AAGGGAGAGGGAGGGAGAAT |
| OSX P2 | CAGCAAATGGAGCAGGAAAT | AAGGGAGAGGGAGGGAGAAT |
| RUNX2 P1 | AAGGAGTTTGCAAGCAGAGC | CAACTGAGTGTGTGGCGTTC |
| RUNX2 P2 | GTGGTAGGCAGTCCCACTTTA | AGAAAGTTTGCACCGCACTTG |
| GAPDH Chip | CGGGATTGTCTGCCCTAAT | GCACGGAAGGTCACGATGT |
| **Primers for negative control siNC and target siRNAs or asoON** | | |
| asoNC | TTCTCCGAACGTGTCACGT | ACGTGACACGTTCGGAGAA |
| asoODIR1-1 | TCTTGATGGCACAGCAGCAG | CTGCTGCTGTGCCATCAAGA |
| asoODIR1-2 | GACAAGAAGAGTTGAAAAGG | CCTTTTCAACTCTTCTTGTC |
| siNC | UUCUCCGAACGUGUCACGUTT | ACGUGACACGUUCGGAGAATT |
| siODIR1-1 | GCAAACGGAAGAGCAGCAATT | UUGCUGCUCUUCCGUUUGCTT |
| siODIR1-2 | GCAUAGAGAUGGCCUUCUUTT | AAGAAGGCCAUCUCUAUGCTT |
| siODIR1-3 | GAGGAAGCCACAUGUAGCUUTT | AAGCUACAUGUGGCUUCCUCTT |
| siZNF518A-1 | GUAGAGUAUUUGACAAUCATT | UGAUUGUCAAAUACUCUACTT |
| siZNF518A-2 | GAAGAUGCCUAGAAUUUCATT | UGAAAUUCUAGGCAUCUUCTT |
| siZNF518A-3 | GUGGCACCAUGGCGAAUUATT | UAAUUCGCCAUGGUGCCACTT |
| siFBXO25-1 | GCUCUACCCUCUGCAUUCUTT | AGAAUGCAGAGGGUAGAGCTT |
| siFBXO25-2 | CCACCACAAUCCUCGCUUATT | UAAGCGAGGAUUGUGGUGGTT |
| siFBXO25-3 | GGAGUAGGGAAGUCUGUAUTT | AUACAGACUUCCCUACUCCTT |
| siCUL3-1 | GAAGGAAUGUUUAGGGAUATT | UAUCCCUAAACAUUCCUUCTT |
| siCUL3-2 | GCACUGCCUUGACAAAUCATT | UGAUUUGUCAAGGCAGUGCTT |
| siCUL3-3 | CAAUGACCGUCUCUUUAAATT | UUUAAAGAGACGGUCAUUGTT |
| siBARD1-1 | GAGAGACUUUGCUCCAUAUTT | AUAUGGAGCAAAGUCUCUCTT |
| siBARD1-2 | CAGUGAGCUUGCAGUAAUUTT | AAUUACUGCAAGCUCACUGTT |
| siBARD1-3 | GACUCAGACCAUCAAUACATT | UGUAUUGAUGGUCUGAGUCTT |
| **Digoxin tag probe sequences for ODIR1** | | |
| ODIR1 probe 1 | GTGTCTTGAGAAATGTAACTCAGGGTTGATGCTCTTCCGT |  |
| ODIR1 probe 2 | TGTCACATCCTACTTTCATTAGGATGGAACTCTGAAGTGT |  |
| ODIR1 probe 3 | CTAATGTTTAATACAAACCAATTCACTTCCCATCAGTTTC |  |

Abbreviations: RUNX2, Runt related transcription factor 2; OSX, Osterix/Sp7; OPN, Osteopontin; OCN, Osteocalcin; ZNF518A, Zinc finger protein 518A; CUL3, Cullin 3; FBXO25, F-box protein 25; BARD1, BRCA1 associated RING domain 1 ; ALP, Alkaline phosphatase; aso, antisense oligonucleotide.
